# Superalkali Coated Rydberg Molecules

**DOI:** 10.3389/fchem.2022.880804

**Published:** 2022-04-13

**Authors:** Nikolay V. Tkachenko, Pavel Rublev, Alexander I. Boldyrev, Jean-Marie Lehn

**Affiliations:** ^1^ Department of Chemistry and Biochemistry, Utah State University, Logan, UT, United States; ^2^ Laboratoire de Chimie Supramoléculaire, Institut de Science et d’Ingénierie, Supramoléculaires Université de Strasbourg, Strasbourg, France

**Keywords:** cryptands, cryptatium, superalkalis, Rydberg molecules, ionization potential (IP)

## Abstract

A series of complexes of Na, K, NH_4_, and H_3_O with [bpy.bpy.bpy]cryptand, [2.2.2]cryptand, and spherical cryptand were investigated *via* DFT and ab initio methods. We found that by coating Rydberg molecules with the “organic skin” one could further decrease their ionization potential energy, reaching the values of ∼1.5 eV and a new low record of 1.3 eV. The neutral cryptand complexes in this sense possess a weakly bounded electron and may be considered as very strong reducing agents. Moreover, the presence of an organic cage increases the thermodynamic stability of Rydberg molecules making them stable toward the proton detachment.

## Introduction

**SCHEME 1:**
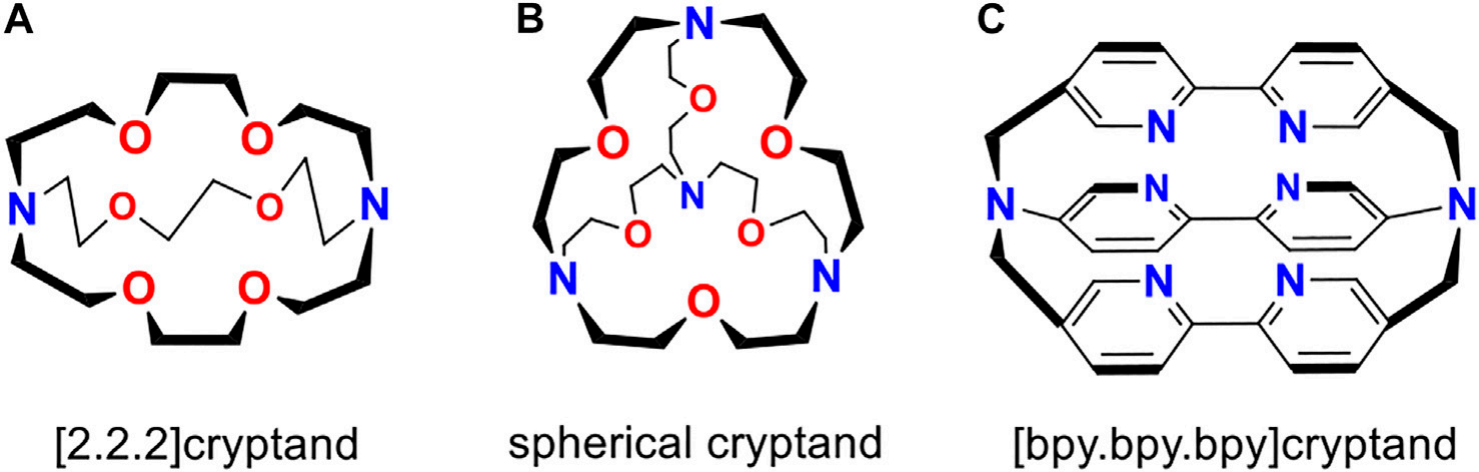
The structures of organic cages considered in this work. [2.2.2]cryptand **(A)**, spherical cryptand **(B)**, and [bpy.bpy.bpy]cryptand **(C)** are shown.

The [2.2.2]cryptand and spherical cryptand (Scheme 1A, B) invented by Lehn (Lehn, 1977), have been a subject of both theoretical (Elroby et al., 2006; Elroby, 2009; Puchta et al., 2019; Isaeva et al., 2021; Ćoćić et al., 2021; Ariyarathna, 2022) and experimental (Lehn, 1977; Lehn, 1978; Lehn, 1979; Lehn, 1980; Echegoyen et al., 1991; Arnaud-Neu et al., 2002; Cram et al., 2002; Izatt et al., 2002; Miyamoto et al., 2002; Badjić et al., 2011; Chung et al., 2020) studies for decades. The discovery of those fascinating compounds opened a huge field of supramolecular chemistry. Their unique guest particle selectivity and extremely low ionization potentials of neutral alkali-metal complexes (Cram and Lein, 1985; Huang et al., 1988; Kim et al., 1999) found an application in synthetic organic and inorganic chemistry. In particular, a huge number of multiply-charged Zintl anions with unusual structures have been synthesized using the popular [K⊂[2.2.2]cryptand] complex (Sun et al., 2018; Tkachenko et al., 2020; Wang et al., 2020).

Firstly, introduced by Gutsev and Boldyrev (Gutsev and Boldyrev, 1982), the family of superalkalis has been growing significantly. Despite the initially proposed M_k+1_L family, where M is an alkali atom and L is an electronegative atom of valence k, other superalkalis have been proposed and synthesized. Along with other inorganic binuclear superalkali (Tong et al., 2009), the definition of superalkalis was extended to polynuclear species such as polynuclear aromatic superalkalis (Sun et al., 2013; Parida et al., 2018), superalkali cations (Tong et al., 2011; Tong et al., 2012a; Tong et al., 2012b; Hou et al., 2013), organo-Zintl clusters (Giri et al., 2016; Reddy and Giri, 2016). Another family of compounds with low ionization potential is Rydberg molecules. Vivid examples of Rydberg molecules are NH_4_ and H_3_O neutral species, whose unpa`ired electron occupies a diffuse orbital around the molecule. It has been shown that such Rydberg molecules are not long-living particles. Although the *T*_*d*_ structure of NH_4_ radical is a local minimum, it is only a metastable molecule and undergoes a dissociation into NH_3_ and H* radical species (Herzberg, 1981; Signorell et al., 1997). Similar behavior is found for H_3_O neutral species (Luo and Jungen, 1999; Melin et al., 2005). It has been shown before that cryptand compounds can bind both NH_4_^+^ and H_3_O^+^ cations with a great selectivity (Cram et al., 1985; Behr et al., 2002; Junk, 2008). Thus, it will be interesting to investigate the electronic properties of neutral [R⊂cryptand] (R = NH_4_, H_3_O) complexes, since the organic coating could stabilize the Rydberg molecules and decrease their ionization potential as it was observed for alkali metal complexes (Cram and Lein, 1985; Huang et al., 1988; Kim et al., 1999). In this work, we investigate the electronic properties of coated Rydberg molecules *via* DFT and *ab initio* methods and compare their properties with alkali-metal cryptand complexes.

## Computational Methods

All structures were optimized using Perdew–Burke-Ernzerhof (PBE0) (Perdew et al., 1996) and Tao-Perdew-Staroverov-Scuseria (TPSSh) (Staroverov et al., 2003) hybrid functionals using def2-SVP basis set (Weigend and Ahlrichs, 2005). The frequency calculations were performed at the same level of theory. No imaginary frequencies were present, showing that the optimized structures are at local minima on the given PES. Ionization potentials were calculated at three different levels of theory. In particular, the single-point calculations at optimized geometry using DFT functionals (PBE0 and TPSSh) and a moderately large basis set def2-TZVPPD (Weigend and Ahlrichs, 2005) were carried out. In addition, single-point calculations using MP2 level of theory with cc-pvdz (C, N, O atoms) and aug-cc-pvdz (H, K, Na atoms) basis sets (Dunning, 1989; Kendall et al., 1992; Hill and Peterson, 2017) were performed. For convenience, we will denote this combination of basis functions as *Basis-1*. Due to the large values of spin contamination, the [bpy.bpy.bpy]cryptand complexes were calculated using ROHF-MBPT2 formalism (Lauderdale et al., 1991; Lauderdale et al., 1992). The vertical ionization potential (VIP) was calculated as the energy difference between the optimized neutral complex and the cation in the geometry of the neutral complex. The adiabatic ionization potential (AIP) was calculated as the energy differences between an optimized neutral cluster and an optimized cation. The natural charge distribution was calculated via NBO method as implemented in NBO7 software (Glendening et al., 2019). The topology analysis of electron localization function (ELF) (Silvi and Savin, 1994) was performed with the Multiwfn program (Lu and Chen, 2012). All calculations were performed with Gaussian 16 program (Frisch et al., 2016). The visualization of SOMO orbitals and geometries of the investigated species were performed using IboView software (Knizia, 2013; Knizia and Klein, 2015).

## Results and Discussion

The neutral [Na⊂[bpy.bpy.bpy]cryptand] was firstly synthesized in 1991 by Lehn and coworkers (Echegoyen et al., 1991) through the electrochemical reduction of [Na^+^⊂[bpy.bpy.bpy]cryptand] cation. This approach potentially can be used for the synthesis of superalkali cryptand complexes with Rydberg molecules. To investigate the electronic properties of such species, we chose three different organic cages ([2.2.2]cryptand, [bpy.bpy.bpy]cryptand, and spherical cryptand) that are very promising candidates for the capturing of NH_4_ and H_3_O species. The structures of those cages are given in Scheme 1. For the comparison of ionization potentials, two alkali metal complexes were also considered. In particular [Na⊂[bpy.bpy.bpy]cryptand] was chosen as the first synthesized cryptand-superalkali species, and [K⊂[2.2.2]cryptand] was chosen as one of the most popular examples of alkali metal macrocyclic complex.

**FIGURE 1:**
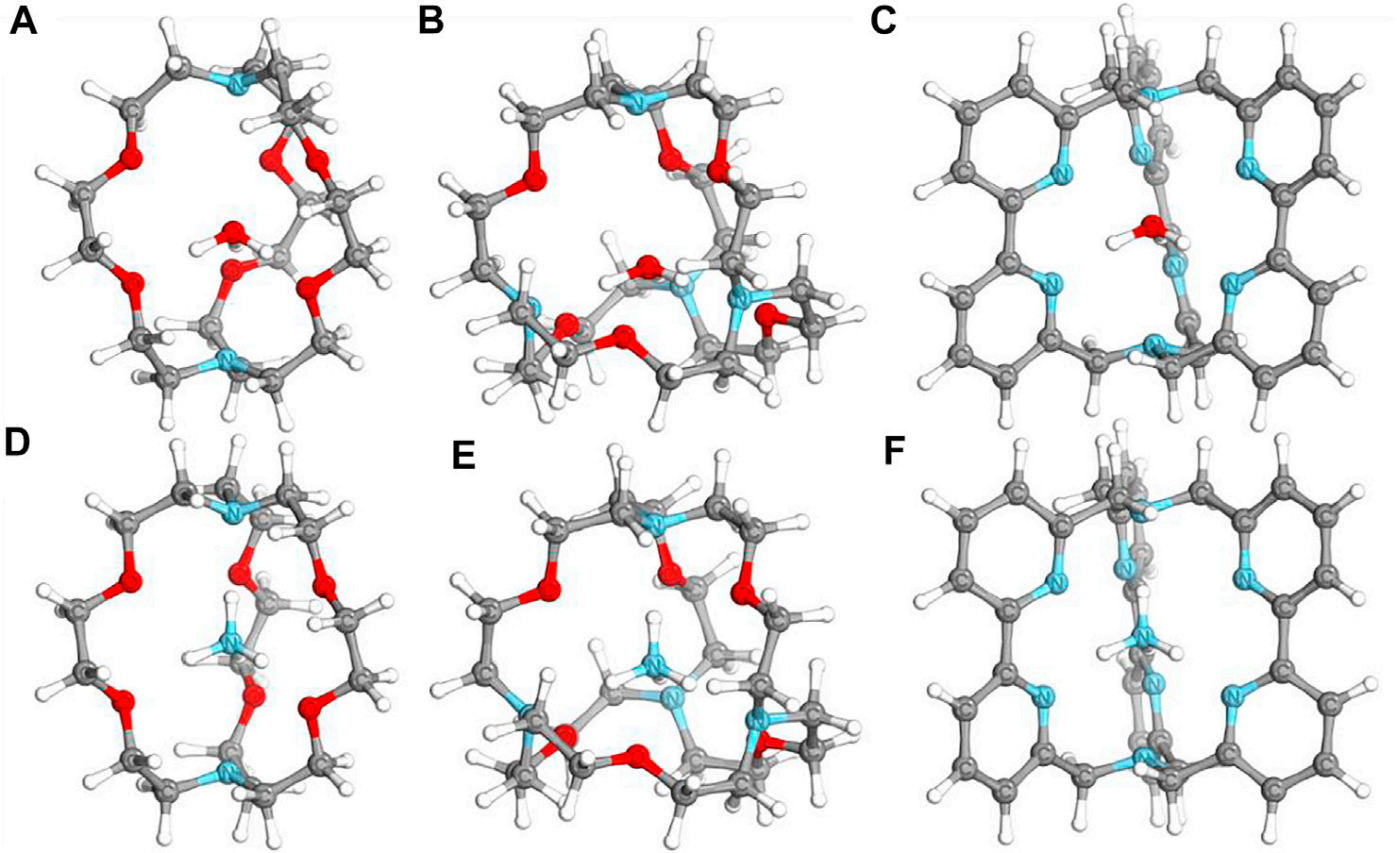
Structures of optimized neutral complexes of NH_4_ and H_3_O. **(A)** [H_3_O⊂[2.2.2]cryptand]; **(B)** [H_3_O⊂spherical cryptand]; **(C)** [H_3_O⊂[bpy.bpy.bpy]cryptand]; **(D)** [NH_4_⊂[2.2.2]cryptand]; **(E)** [NH_4_⊂spherical cryptand]; **(F)** [NH_4_⊂[bpy.bpy.bpy]cryptand].

The geometries of neutral and cationic complexes were optimized with two different DFT hybrid functionals. It was shown before that PBE0 and TPSSh functionals can provide accurate geometries for macrocyclic and cryptand complexes (Tkachenko et al., 2019). The optimized geometries are consistent within two methods, showing the functional independence of the results. The geometries of neutral species are only slightly distorted from the geometries of cationic species, showing that the additional electron of neutral complexes does not participate in a significant bonding formation process. The optimized structures of selected neutral species are given in Figure 1. Cartesian coordinates of all optimized structures are provided in the Supporting Information file (Supplementary Table S1). The natural charge distribution of neutral species showed that the negative charge is mainly distributed over the oxygen and nitrogen atoms of the organic ligand, while the central unit (either H_3_O or NH_4_ species) formally possesses a +1 positive charge. In particular 0.757–0.806 and 0.794–0.876 positive natural charges on H_3_O and NH_4_ molecules, respectively, were found in investigated complexes. This might be one of the key reasons for the stabilization of those Rydberg molecules, which are thermodynamically unstable toward dissociation of a proton in their naked form.

To illustrate the enhanced stability of encapsulated neutral molecules we performed calculations of dissociation energies for both naked and coated species. The reaction that was considered is a dissociation of a proton from the central unit with a formal reaction: $AH^{•}\to A+H^{•}$ . Energies were calculated using the following expression: $\Delta G_{r}=\Delta G\left(A\right)+\Delta G\left(H^{•}\right)-\Delta G\left(AH^{•}\right)$ . The results are shown in Table 1. As we can observe, the dissociation of naked H_3_O and NH_4_ occurs with a significant release of energy (21.9 and 13.0 kcal/mol, respectively). While the dissociation of the same species coated by cryptand complexes is energetically not favorable for most of the compexes (Table 1). Such a difference in $\Delta G_{r}$ values can lead us to the conclusion that [R⊂cryptand] complexes are thermodynamically more stable species, which may open the possibility of their fabrication.

**TABLE 1:** Free energies [kcal/mol] for the dissociation reaction of hydrogen radical from the central unit of investigated species calculated at TPSSh/def2-TZVPPD//TPSSh/def2-SVP level.

Species	ΔG_r_	Species	ΔG_r_
NH_4_	−12.98	H_3_O	−21.94
[NH_4_⊂spherical cryptand]	24.61	[H_3_O⊂spherical cryptand]	−6.43
[NH_4_⊂[2.2.2]cryptand]	17.10	[H_3_O⊂[2.2.2]cryptand]	−26.00
[NH_4_⊂[bpy.bpy.bpy]cryptand]	52.98	[H_3_O⊂[bpy.bpy.bpy]cryptand]	32.55

Interestingly, for both NH_4_ and H_3_O, a significant decrease in ionization potentials was found after encapsulating the corresponding Rydberg molecules into organic cages. Particularly, the naked NH_4_ and H_3_O molecules possess 4.57 and 5.55 eV VIP, respectively. Whereas the NH_4_ and H_3_O encapsulated in [2.2.2]cryptand and spherical cryptand possess ionization potentials about 3–4 eV lower than the naked species (Table 2). Interestingly [bpy.bpy.bpy]cryptand systems show larger IPs by ∼1.1 eV. A similar but not so pronounced pattern was found for alkali metals encapsulated in the [bpy.bpy.bpy]cryptand. The nature of such an increase in IPs is discussed below and related to the presence of a diffuse SOMO orbital in the system. We note, that the obtained IPs for NH_4_ and H_3_O species are even lower than IPs of [K⊂[2.2.2]cryptand] which was shown before to be a superalkali with record low ionization potential (Tkachenko et al., 2019).

**TABLE 2:** Values of VIP and AIP [eV] obtained at MP2/*Basis-1* level of theory.

Species	AIP	VIP	Species	AIP	VIP
Na	N/A	4.961	[NH_4_⊂spherical cryptand]	1.358	1.389
K	N/A	4.072	[NH_4_⊂[2.2.2]cryptand]	1.308	1.381
NH_4_	4.429	4.566	[NH_4_⊂[bpy.bpy.bpy]cryptand]	2.385	2.582
H_3_O	5.310	5.552	[H_3_O⊂spherical cryptand]	1.379	1.696
[Na⊂[bpy.bpy.bpy]cryptand]	2.440	2.729	[H_3_O⊂[2.2.2]cryptand]	1.362	1.676
[K⊂[2.2.2]cryptand]	1.387	1.612	[H_3_O⊂[bpy.bpy.bpy]cryptand]	2.501	2.729

Similar results were obtained using PBE0 and TPSSh functionals with def2-TZVPPD basis set. Although the values of IPs are slightly higher than it was obtained for the MP2 method, the main trends preserve the same (Table 3).

**TABLE 3:** Values of VIP and AIP [eV] obtained using PBE0 and TPSSh functionals with def2-TZVPPD basis set.

Species	PBE0		TPSSh	
	AIP	VIP	AIP	VIP
K	N/A	4.370	N/A	4.233
Na	N/A	5.280	N/A	5.152
NH_4_	4.417	4.584	4.313	4.462
H_3_O	5.384	5.964	5.343	5.606
[NH_4_⊂spherical cryptand]	1.440	1.519	1.400	1.479
[NH_4_⊂[2.2.2]cryptand]	1.445	1.537	1.403	1.504
[NH_4_⊂[bpy.bpy.bpy]cryptand]	3.060	3.220	3.214	3.359
[H_3_O⊂ spherical cryptand]	1.457	1.780	1.388	1.704
[H_3_O⊂[2.2.2]cryptand]	1.387	1.821	1.492	1.638
[H_3_O⊂[bpy.bpy.bpy]cryptand]	3.162	3.323	3.307	3.452
[Na⊂[bpy.bpy.bpy]cryptand]	3.157	3.335	3.298	3.407
[K⊂[2.2.2]cryptand]	1.811	1.830	1.784	1.803

**FIGURE 2:**
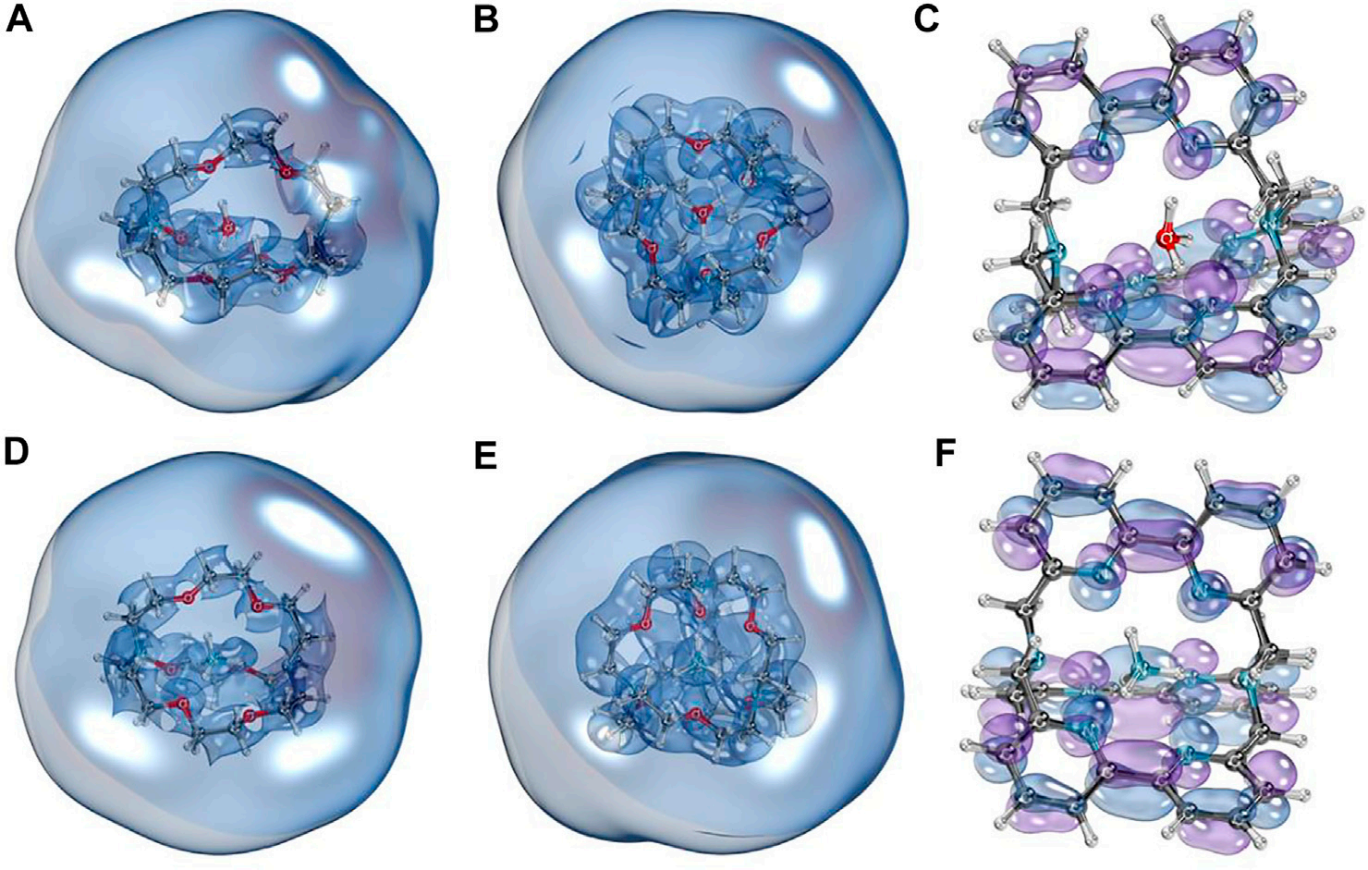
Isosurface plots of singly occupied molecular orbitals of NH_4_ and H_3_O complexes. **(A)** [H_3_O⊂[2.2.2]cryptand]; **(B)** [H_3_O⊂spherical cryptand]; **(C)** [H_3_O⊂[bpy.bpy.bpy]cryptand]; **(D)** [NH_4_⊂[2.2.2]cryptand]; **(E)** [NH_4_⊂spherical cryptand]; **(F)** [NH_4_⊂[bpy.bpy.bpy]cryptand]. A threshold of 100% was used for A, B, D, E to illustrate the diffuse nature of those orbitals. For C and F a lower 80% threshold was used for visualization.

To illustrate the diffuse nature of SOMO of investigated species, we plotted the isosurface graphs of corresponding orbitals shown in Figure 2 (the orbitals were obtained from quasi-restricted orbitals formalism). We can see that for [2.2.2]cryptand and spherical cryptand complexes (Figures 2A,B,D,E), SOMO orbitals have a diffuse nature and surround the whole molecule entirely. In contrast, the unpaired electron of [bpy.bpy.bpy]cryptand complexes sit on the antibonding orbital of a π-conjugated system (Figures 2C,F). Isosurface plots of SOMO visualized with a different contour value can be found in the supporting information file (Supplementary Figure S1). Such an interesting difference in SOMO can be explained by the fact that different organic cages form different types of complexes with Rydberg molecules. Thus [2.2.2]cryptand and spherical cryptand complexes behave as electrides, possessing an electron density outside of the molecule, whereas [bpy.bpy.bpy]cryptand complexes form an ionic molecular compound bearing a negative charge entirely on the organic ligand. Such behavior can also be explained by the possibility of bipyridine molecules to form stable anionic species, that were experimentally isolated before (Bock et al., 1999; Gore-Randall et al., 2009). To further show the differences between the two types of complexes we performed an ELF basins analysis. The basins laying outside of the molecule were found for [2.2.2]cryptand and spherical cryptand complexes (Supplementary Figure S2). The integration of the electron density within the volume of the found ELF basins resulted in 0.3–0.6 |e| basins occupancy. In turn, no outside lying ELF basins were found for [bpy.bpy.bpy]cryptand complexes. Thus [2.2.2]cryptand and spherical cryptand complexes demonstrate an electride nature which is the reason for their lower IP values in comparison to [bpy.bpy.bpy]cryptand complexes.

## Conclusion

In this work we investigated the electronic properties of Rydberg molecules coated with cryptand organic cages. We showed that it is possible to significantly decrease the values of the ionization potentials by covering Rydberg molecules with an “organic skin.” In particular, we found that the IP could be decreased, reaching the values of ∼1.5 eV and a new low record of 1.3 eV (at MP2/*Basis-1* level). In addition, the coating ligand can increase the thermodynamic stability of a Rydberg molecule, opening an opportunity to obtain such strong reducing agents in the experiment.

## Data Availability

The additional data that support the findings of this study are available from the corresponding author on a reasonable request.
