# Supplementary material for: Superalkali Coated Rydberg Molecules
Source: Front Chem. 2022 Apr 13;10:880804. doi: 10.3389/fchem.2022.880804 (PMC9043523; doi:10.3389/fchem.2022.880804)
Supplement: Supplementary file 1 [file DataSheet1.pdf]

Supplementary Material

1 Supplementary Figures

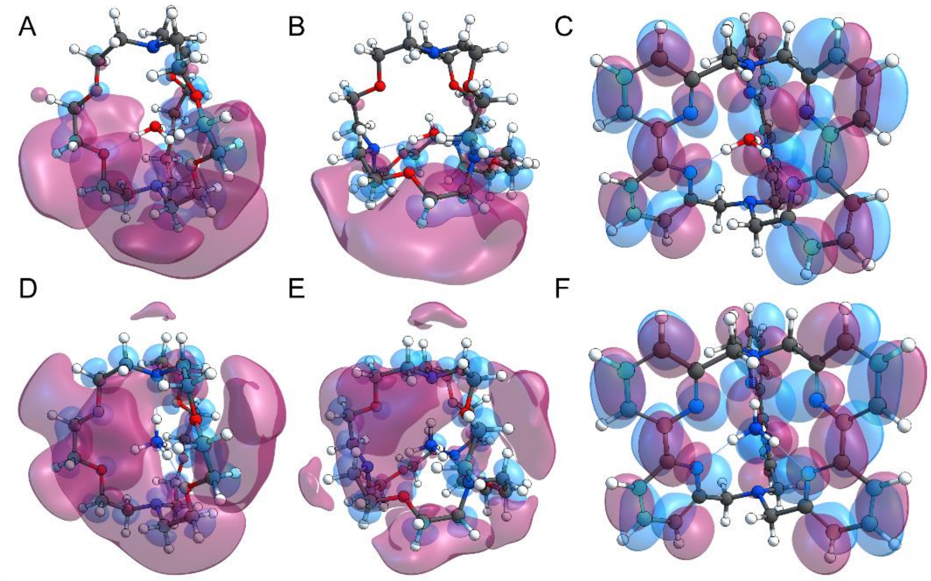

Figure S1. Isosurface plots of singly occupied molecular orbitals of NH_4 and H_3O complexes. (A) [H_3O⊂[2.2.2]cryptand]; (B) [H_3O⊂spherical cryptand]; (C) [H_3O⊂[bpy.bpy.bpy]cryptand]; (D) [NH_4⊂[2.2.2]cryptand]; (E) [NH_4⊂spherical cryptand]; (F) [NH_4⊂[bpy.bpy.bpy]cryptand]. Contour value was set to 0.013 a.u.

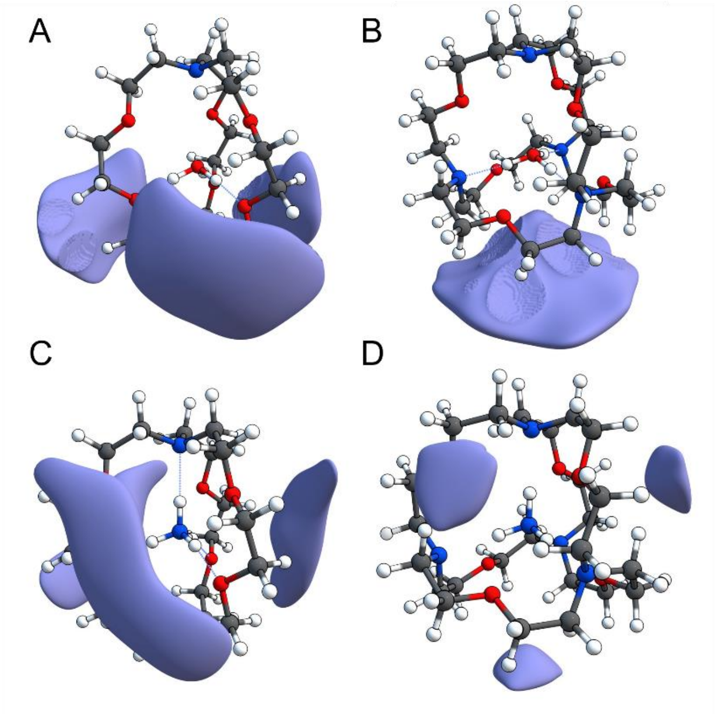

Figure S2. Isosurface plots of ELF basins located outside of the molecule for NH_4 and H_3O complexes calculated at at TPSSh/def2-TZVPPD//TPSSh/def2-SVP level. (A) [H_3O⊂[2.2.2]cryptand]; (B) [H_3O⊂spherical cryptand]; (C) [NH_4⊂[2.2.2]cryptand]; (D) [NH_4⊂spherical cryptand]; Contour value was set to 0.05 a.u.

2 Supplementary Tables

Table S1. Cartesian coordinates of optimized molecules and complexes studied in this work.

NH_4	TPSSh/def2-SVP (0 imaginary Frequencies)
	N 0.013009000 -0.013009000 0.013008000<br>H -0.593694000 0.593694000 0.556937000<br>H 0.556960000 0.593688000 -0.593679000<br>H -0.593688000 -0.556960000 -0.593679000
NH_4^+	TPSSh/def2-SVP (0 imaginary Frequencies)
	N 0.000000000 0.000000000 0.000000000<br>H -0.604679000 0.604679000 0.604679000<br>H 0.604679000 0.604679000 -0.604679000<br>H -0.604679000 -0.604679000 -0.604679000<br>H 0.604679000 -0.604679000 0.604679000
[NH_4⊂spherical cryptand]	TPSSh/def2-SVP (0 imaginary Frequencies)
	N 1.833387000 -1.861347000 -1.672154000<br>N -1.736050000 1.726673000 -1.903906000<br>N 1.748746000 1.852494000 1.769859000<br>N 2.120710000 1.066473000 2.949675000<br>N 0.956538000 0.664816000 3.842321000

	O	-0.082357000	0.114180000	3.057766000
	N	-1.164155000	-0.376794000	3.823699000
	N	-2.279360000	-0.845557000	2.901719000
	N	-1.846170000	-1.717670000	1.806256000
	N	-2.964317000	-2.151281000	0.963631000
	N	-3.856405000	-1.027487000	0.458942000
	O	-3.062477000	0.009000000	-0.082653000
	N	-3.820081000	1.049564000	-0.666846000
	N	-2.895643000	2.167731000	-1.123497000
	N	-1.034515000	-2.843409000	2.277550000
	N	-0.567845000	-3.794765000	1.186345000
	O	-0.010425000	-3.060150000	0.115053000
	N	0.539828000	-3.876807000	-0.898994000
	N	1.013116000	-3.013049000	-2.058071000
	N	2.948497000	-2.239165000	-0.799198000
	N	3.848706000	-1.087448000	-0.378864000
	O	3.062362000	-0.007858000	0.083799000
	N	3.827564000	1.068263000	0.588148000
	N	2.911444000	2.224502000	0.958721000
	N	-2.114312000	0.855934000	-3.020631000
	N	-0.953583000	0.380092000	-3.880739000
	O	0.083099000	-0.114899000	-3.057137000
	N	1.160742000	-0.670035000	-3.784221000
	N	2.272855000	-1.077108000	-2.829765000
	N	0.914014000	3.008098000	2.110287000
	N	0.509712000	3.874092000	0.927045000
	O	0.012079000	3.059918000	-0.115743000
	N	-0.481372000	3.796767000	-1.216450000
	N	-0.893173000	2.847081000	-2.330985000
	H	1.425049000	3.672461000	2.850357000
	H	-0.004438000	2.624232000	2.580332000
	H	-0.275482000	4.580924000	1.272807000
	H	1.358536000	4.485975000	0.556737000
	H	-1.325538000	4.440500000	-0.892322000
	H	0.308078000	4.470267000	-1.615047000
	H	0.021842000	2.422065000	-2.771344000
	H	-1.400283000	3.457560000	-3.118617000
	H	-2.607941000	-0.030943000	-2.594420000
	H	-2.845704000	1.361862000	-3.698364000
	H	-1.330816000	-0.423030000	-4.550417000
	H	-0.564773000	1.191008000	-4.531369000
	H	0.804887000	-1.528996000	-4.390550000
	H	1.575092000	0.079883000	-4.492502000
	H	2.744915000	-0.159554000	-2.446201000
	H	3.038311000	-1.631698000	-3.427043000
	H	-3.515893000	2.898984000	-1.698643000
	H	-2.514078000	2.681064000	-0.227529000
	H	-4.529745000	1.474072000	0.076057000

	H	-4.427127000	0.649969000	-1.505846000
	H	-4.505510000	-0.624893000	1.264353000
	H	-4.527386000	-1.447463000	-0.321539000
	H	-2.537735000	-2.665964000	0.088969000
	H	-3.618888000	-2.879498000	1.503509000
	H	0.123933000	-2.624941000	-2.578244000
	H	1.561208000	-3.679975000	-2.768733000
	H	-0.228767000	-4.579604000	-1.287661000
	H	1.363778000	-4.493044000	-0.482526000
	H	-1.396604000	-4.433498000	0.815834000
	H	0.194676000	-4.473043000	1.627083000
	H	-1.587455000	-3.451400000	3.035719000
	H	-0.142380000	-2.424160000	2.767537000
	H	3.597792000	-3.010119000	-1.283222000
	H	2.518344000	-2.684523000	0.110983000
	H	4.500689000	-0.750540000	-1.211633000
	H	4.516674000	-1.453099000	0.430961000
	H	4.540166000	1.430268000	-0.184397000
	H	4.431886000	0.728775000	1.455099000
	H	2.533474000	2.671425000	0.026383000
	H	3.537246000	2.992610000	1.476998000
	H	2.608402000	0.146549000	2.592268000
	H	2.855282000	1.617350000	3.587762000
	H	0.572087000	1.525562000	4.428305000
	H	1.328474000	-0.086557000	4.572240000
	H	-1.572859000	0.427174000	4.473565000
	H	-0.814893000	-1.190604000	4.492874000
	H	-3.049107000	-1.347794000	3.538480000
	H	-2.744189000	0.044354000	2.450277000
	H	-0.584513000	0.580486000	-0.640467000
	H	0.587570000	0.622362000	0.594845000
	H	-0.620520000	-0.578708000	0.607312000
	H	0.615724000	-0.626086000	-0.563641000
	N	-0.000418000	-0.000521000	-0.000484000
[NH_4^+⊂spherical cryptand]	TPSSh/def2-SVP (0 imaginary Frequencies)			
	N	-2.398432000	1.790481000	-0.814104000
	N	1.536165000	-0.890304000	-2.542774000
	N	-1.042903000	-2.501702000	1.506773000
	N	-1.266964000	-2.079734000	2.893278000
	N	-0.000989000	-1.702971000	3.649926000
	O	0.741090000	-0.774020000	2.885335000
	N	1.854269000	-0.232420000	3.567748000
	N	2.677664000	0.606551000	2.600584000
	N	1.905248000	1.601908000	1.850054000
	N	2.745287000	2.415867000	0.965601000
	N	3.689684000	1.614974000	0.080263000
	O	2.958107000	0.611366000	-0.594794000

	N	3.708933000	-0.085842000	-1.568701000
	N	2.886232000	-1.251841000	-2.098634000
	N	1.044681000	2.415678000	2.714904000
	N	0.256859000	3.494649000	1.985225000
	O	-0.423680000	2.915950000	0.889671000
	N	-1.316275000	3.799090000	0.240386000
	N	-1.851965000	3.135585000	-1.020573000
	N	-3.348065000	1.733729000	0.301953000
	N	-3.977229000	0.365798000	0.525390000
	O	-2.957832000	-0.610974000	0.595713000
	N	-3.420969000	-1.894670000	0.964242000
	N	-2.283190000	-2.897183000	0.831423000
	N	1.531153000	0.263823000	-3.447471000
	N	0.154520000	0.647747000	-3.971622000
	O	-0.740746000	0.773501000	-2.884911000
	N	-2.007812000	1.286457000	-3.245146000
	N	-2.941822000	1.208634000	-2.045641000
	N	0.009230000	-3.516692000	1.390574000
	N	0.245980000	-4.019969000	-0.026428000
	O	0.423375000	-2.915574000	-0.890740000
	N	0.813061000	-3.272986000	-2.201718000
	N	0.797312000	-2.034385000	-3.086484000
	H	-0.200556000	-4.407666000	2.023770000
	H	0.943967000	-3.062632000	1.750399000
	H	1.149272000	-4.661173000	-0.014632000
	H	-0.590301000	-4.653210000	-0.382743000
	H	1.810884000	-3.754063000	-2.185577000
	H	0.109947000	-4.011154000	-2.635263000
	H	-0.248943000	-1.719421000	-3.211607000
	H	1.180855000	-2.340607000	-4.085455000
	H	1.932990000	1.120983000	-2.887858000
	H	2.184966000	0.100819000	-4.333343000
	H	0.251890000	1.607567000	-4.516143000
	H	-0.229850000	-0.094204000	-4.699016000
	H	-1.906988000	2.323775000	-3.620837000
	H	-2.455700000	0.692034000	-4.065696000
	H	-3.147657000	0.147351000	-1.843733000
	H	-3.900213000	1.688858000	-2.345110000
	H	3.478441000	-1.727374000	-2.912241000
	H	2.778901000	-1.987842000	-1.288564000
	H	4.639916000	-0.496177000	-1.130378000
	H	4.014982000	0.603412000	-2.380264000
	H	4.513516000	1.159498000	0.664354000
	H	4.157852000	2.314351000	-0.640090000
	H	2.072798000	2.990725000	0.312456000
	H	3.367350000	3.142214000	1.535044000
	H	-1.021697000	3.045616000	-1.736080000
	H	-2.604739000	3.827575000	-1.460212000

	H -0.800585000 4.733066000 -0.057899000<br>H -2.132810000 4.091586000 0.929608000<br>H 0.912068000 4.317109000 1.636507000<br>H -0.458933000 3.939800000 2.704052000<br>H 1.623078000 2.920879000 3.520535000<br>H 0.325526000 1.736011000 3.194750000<br>H -4.181376000 2.460849000 0.176618000<br>H -2.798653000 2.007927000 1.214267000<br>H -4.697661000 0.108868000 -0.276027000<br>H -4.552679000 0.404899000 1.471203000<br>H -4.244576000 -2.222978000 0.300079000<br>H -3.830595000 -1.871965000 1.993320000<br>H -2.052447000 -3.009692000 -0.237904000<br>H -2.664217000 -3.875569000 1.200720000<br>H -1.921699000 -1.196591000 2.865342000<br>H -1.783011000 -2.865518000 3.489115000<br>H 0.620156000 -2.591068000 3.879661000<br>H -0.301766000 -1.263915000 4.621518000<br>H 2.505703000 -1.037223000 3.961633000<br>H 1.515829000 0.360081000 4.440520000<br>H 3.498033000 1.074404000 3.189575000<br>H 3.136855000 -0.072714000 1.867654000<br>H 0.516863000 -0.300279000 -0.855419000<br>H -0.350906000 -0.842267000 0.508595000<br>H 0.641618000 0.539468000 0.623097000<br>H -0.807039000 0.602616000 -0.273530000<br>N 0.000114000 -0.000148000 0.000705000
[NH_4⊂[2.2.2]cryptand]	TPSSh/def2-SVP (0 imaginary Frequencies)
	O 1.386369000 0.599328000 -2.469072000<br>O -1.406773000 0.204147000 -2.561586000<br>O -1.406655000 -2.320659000 1.103981000<br>O 1.386644000 -2.437664000 0.715870000<br>O 1.386225000 1.838998000 1.753269000<br>O -1.407109000 2.117148000 1.457267000<br>N -2.978615000 -0.000354000 0.000151000<br>N 3.025468000 -0.000115000 0.000003000<br>N 2.755657000 2.069545000 1.432235000<br>H 3.240374000 2.569180000 2.301202000<br>H 2.829536000 2.764284000 0.572316000<br>N 3.476794000 0.759849000 1.173447000<br>H 4.574539000 0.969895000 1.102107000<br>H 3.334511000 0.132844000 2.065929000<br>N 2.755630000 0.205509000 -2.508301000<br>H 3.240466000 0.707897000 -3.375599000<br>H 2.829048000 -0.886646000 -2.679801000<br>N 3.477061000 0.636115000 -1.244784000<br>H 4.574750000 0.468996000 -1.391076000

	H	3.335168000	1.722587000	-1.148019000
	N	2.755970000	-2.275015000	1.076371000
	H	3.240614000	-3.277420000	1.074769000
	H	2.829587000	-1.877543000	2.107975000
	N	3.477333000	-1.396150000	0.071659000
	H	4.574972000	-1.438890000	0.289843000
	H	3.335783000	-1.855726000	-0.917613000
	N	0.695578000	3.023652000	2.140974000
	H	0.680028000	3.747661000	1.302723000
	H	1.234000000	3.489904000	2.998033000
	N	-0.712412000	2.682862000	2.555706000
	H	-0.694469000	1.975600000	3.409432000
	H	-1.231019000	3.612934000	2.888886000
	N	0.695650000	0.343221000	-3.688906000
	H	0.680068000	-0.744657000	-3.897204000
	H	1.233979000	0.852642000	-4.521091000
	N	-0.712353000	0.872690000	-3.600821000
	H	-0.694496000	1.965656000	-3.415104000
	H	-1.231153000	0.696188000	-4.572778000
	N	0.695923000	-3.366398000	1.547164000
	H	0.680243000	-3.003262000	2.593579000
	H	1.234373000	-4.341739000	1.521730000
	N	-0.711966000	-3.554788000	1.044305000
	H	-0.693885000	-3.940015000	0.004757000
	H	-1.230738000	-4.308602000	1.682785000
	N	-2.767317000	0.585928000	-2.458773000
	H	-3.281059000	0.383521000	-3.426782000
	H	-2.848884000	1.676072000	-2.273581000
	N	-3.454662000	-0.227783000	-1.379198000
	H	-4.555844000	-0.042252000	-1.430311000
	H	-3.296454000	-1.287817000	-1.624822000
	N	-2.767323000	1.836199000	1.737100000
	H	-3.281614000	2.775257000	2.046278000
	H	-2.847916000	1.130449000	2.588447000
	N	-3.454897000	1.307834000	0.492850000
	H	-4.556025000	1.259076000	0.679348000
	H	-3.297112000	2.050573000	-0.302425000
	N	-2.767229000	-2.422847000	0.722075000
	H	-3.280821000	-3.159881000	1.381582000
	H	-2.848828000	-2.807863000	-0.314495000
	N	-3.454850000	-1.081182000	0.886727000
	H	-4.555987000	-1.218356000	0.751305000
	H	-3.297093000	-0.763826000	1.927620000
	H	0.389639000	0.209252000	-0.946234000
	H	0.390523000	-0.923834000	0.292268000
	H	0.389805000	0.715347000	0.654257000
	H	-1.022641000	-0.000455000	0.000421000
	N	0.022989000	0.000061000	0.000207000

[$NH_4^+ \subset$[2.2.2]cryptand]	TPSSh/def2-SVP (0 imaginary Frequencies)			
	O	-1.399817000	0.794788000	2.413874000
	O	1.425316000	0.515073000	2.534582000
	O	1.425172000	-2.452726000	-0.821268000
	O	-1.399872000	-2.488012000	-0.518666000
	O	-1.399923000	1.693327000	-1.895160000
	O	1.425181000	1.937674000	-1.713301000
	N	3.000133000	-0.000027000	-0.000087000
	N	-3.055529000	-0.000082000	0.000010000
	N	-2.721821000	2.016255000	-1.479873000
	H	-3.240555000	2.556961000	-2.293646000
	H	-2.690135000	2.695886000	-0.606523000
	N	-3.502481000	0.744107000	-1.183918000
	H	-4.579275000	1.012216000	-1.110770000
	H	-3.400258000	0.092628000	-2.064277000
	N	-2.721667000	0.273587000	2.486111000
	H	-3.240330000	0.708031000	3.361281000
	H	-2.689865000	-0.822556000	2.638108000
	N	-3.502504000	0.653137000	1.236464000
	H	-4.579234000	0.455515000	1.432187000
	H	-3.400527000	1.741305000	1.112348000
	N	-2.721722000	-2.289905000	-1.006123000
	H	-3.240427000	-3.265022000	-1.067482000
	H	-2.689986000	-1.873431000	-2.031395000
	N	-3.502502000	-1.397490000	-0.052535000
	H	-4.579249000	-1.468159000	-0.321483000
	H	-3.400445000	-1.834108000	0.951878000
	N	-0.679405000	2.811929000	-2.397620000
	H	-0.579580000	3.590237000	-1.617365000
	H	-1.218012000	3.252825000	-3.257318000
	N	0.690731000	2.357677000	-2.846989000
	H	0.590680000	1.532374000	-3.579243000
	H	1.199395000	3.200924000	-3.354168000
	N	-0.679258000	0.671000000	3.633855000
	H	-0.579447000	-0.393781000	3.918120000
	H	-1.217823000	1.195357000	4.445376000
	N	0.690900000	1.287187000	3.464929000
	H	0.590893000	2.333884000	3.116000000
	H	1.199571000	1.305077000	4.448781000
	N	-0.679368000	-3.482652000	-1.235900000
	H	-0.579574000	-3.196398000	-2.300147000
	H	-1.217966000	-4.447612000	-1.187568000
	N	0.690775000	-3.644500000	-0.617816000
	H	0.590756000	-3.865715000	0.463105000
	H	1.199439000	-4.505466000	-1.094293000
	N	2.738921000	0.992398000	2.313993000
	H	3.292756000	1.045679000	3.271903000

	H 2.712795000 2.019972000 1.900259000<br>N 3.476785000 0.029882000 1.396549000<br>H 4.560174000 0.269755000 1.432541000<br>H 3.357515000 -0.976006000 1.824241000<br>N 2.738665000 1.507736000 -2.016589000<br>H 3.292524000 2.310543000 -2.541854000<br>H 2.712268000 0.635602000 -2.699563000<br>N 3.476756000 1.194472000 -0.724419000<br>H 4.560089000 1.105563000 -0.950353000<br>H 3.357712000 2.067877000 -0.067194000<br>N 2.738765000 -2.500292000 -0.297566000<br>H 3.292598000 -3.356565000 -0.730258000<br>H 2.712567000 -2.655657000 0.799219000<br>N 3.476680000 -1.224547000 -0.672497000<br>H 4.560070000 -1.375684000 -0.482779000<br>H 3.357401000 -1.092033000 -1.757475000<br>H -0.388973000 0.294366000 0.923406000<br>H -0.388953000 -0.946925000 -0.206810000<br>H -0.388941000 0.652542000 -0.716710000<br>H 1.025968000 -0.000043000 -0.000048000<br>N -0.020829000 -0.000011000 -0.000045000
[NH_4⊂[bpy.bpy.bpy]cryptand]	TPSSh/def2-SVP (0 imaginary Frequencies)
	N 2.884922000 -0.000115000 -0.022469000<br>N -2.865407000 0.000115000 0.030277000<br>N -3.293274000 -0.884899000 -1.057031000<br>H -4.401609000 -0.905070000 -1.147828000<br>H -2.974905000 -1.904623000 -0.784172000<br>N -3.287323000 1.386808000 -0.185165000<br>H -4.394229000 1.483235000 -0.133809000<br>H -2.989427000 1.656393000 -1.211973000<br>N -3.267332000 -0.504459000 1.346397000<br>H -4.374012000 -0.580519000 1.428196000<br>H -2.941879000 0.242043000 2.089704000<br>N 3.301857000 0.636087000 1.223479000<br>H 4.409872000 0.735284000 1.285907000<br>H 3.000159000 -0.034054000 2.046301000<br>N 3.278923000 0.766675000 -1.200667000<br>H 4.385085000 0.782108000 -1.332423000<br>H 2.967828000 1.810420000 -1.025340000<br>N 3.293989000 -1.398871000 -0.101826000<br>H 4.401817000 -1.508140000 -0.055581000<br>H 2.981844000 -1.771022000 -1.092281000<br>N -2.697881000 -0.565683000 -2.422842000<br>N -1.369384000 -0.448017000 -2.502764000<br>N -3.532506000 -0.478113000 -3.548815000<br>N -0.777465000 -0.247253000 -3.703836000<br>N -2.933839000 -0.275170000 -4.802792000<br>N -1.551725000 -0.162619000 -4.886742000

	H	-4.616400000	-0.566707000	-3.447723000
	H	-3.548097000	-0.194066000	-5.703093000
	H	-1.078016000	0.019208000	-5.852804000
	N	0.696586000	-0.116740000	-3.726157000
	N	1.320580000	0.182181000	-2.558667000
	N	1.447856000	-0.291484000	-4.912863000
	N	2.651451000	0.320207000	-2.516216000
	N	2.830130000	-0.165022000	-4.870615000
	N	3.456429000	0.146512000	-3.651542000
	H	0.952836000	-0.553033000	-5.849304000
	H	3.424340000	-0.316624000	-5.775153000
	H	4.541046000	0.253343000	-3.583846000
	N	-2.657460000	-1.844749000	1.739496000
	N	-1.329417000	-1.965173000	1.658427000
	N	-3.477864000	-2.871910000	2.234589000
	N	-0.724100000	-3.102060000	2.072252000
	N	-2.865160000	-4.056704000	2.671549000
	N	-1.482717000	-4.176578000	2.596053000
	H	-4.562152000	-2.747843000	2.276914000
	H	-3.468570000	-4.882657000	3.056404000
	H	-0.998203000	-5.102243000	2.910835000
	N	0.750617000	-3.174286000	1.950662000
	N	1.355413000	-2.307087000	1.101138000
	N	1.518028000	-4.108899000	2.684666000
	N	2.684691000	-2.324673000	0.945345000
	N	2.899243000	-4.122958000	2.536668000
	N	3.506022000	-3.215622000	1.652573000
	H	1.037491000	-4.794217000	3.384480000
	H	3.506996000	-4.826338000	3.111227000
	H	4.589012000	-3.200027000	1.513651000
	N	-2.667100000	2.409792000	0.759270000
	N	-1.336997000	2.409986000	0.884201000
	N	-3.484454000	3.353939000	1.402823000
	N	-0.726993000	3.348354000	1.644986000
	N	-2.866858000	4.337760000	2.190615000
	N	-1.482745000	4.342237000	2.312793000
	H	-4.570262000	3.320070000	1.289974000
	H	-3.467560000	5.086422000	2.713187000
	H	-0.994238000	5.088077000	2.941820000
	N	0.749313000	3.289097000	1.751574000
	N	1.358639000	2.124206000	1.418166000
	N	1.515237000	4.396729000	2.185478000
	N	2.689112000	2.004396000	1.501708000
	N	2.897876000	4.283660000	2.257532000
	N	3.508642000	3.067258000	1.910217000
	H	1.032258000	5.342773000	2.434377000
	H	3.503725000	5.136969000	2.572246000

	H	4.592837000	2.945541000	1.955854000
	H	0.401361000	-0.894978000	0.389698000
	H	0.402285000	0.787292000	0.579485000
	H	0.392166000	0.110049000	-0.971284000
	H	-0.987508000	0.000687000	0.008805000
	N	0.045776000	0.000718000	0.002524000
[$NH_4^+ \subset$[bpy.bpy.bpy]cryptand]	TPSSh/def2-SVP (0 imaginary Frequencies)			
	N	0.000000000	0.000000000	2.912045000
	N	0.000000000	0.000000000	-2.892441000
	N	-1.379227000	0.270394000	-3.322810000
	H	-1.473097000	0.217742000	-4.427903000
	H	-1.613519000	1.306277000	-3.028184000
	N	0.455445000	-1.329642000	-3.322810000
	H	0.547979000	-1.384611000	-4.427903000
	H	-0.324510000	-2.050487000	-3.028184000
	N	0.923782000	1.059248000	-3.322810000
	H	0.925119000	1.166869000	-4.427903000
	H	1.938029000	0.744210000	-3.028184000
	N	1.397436000	-0.111532000	3.331935000
	H	1.490652000	-0.198162000	4.436728000
	H	1.900579000	0.827020000	3.045179000
	N	-0.795308000	-1.154449000	3.331935000
	H	-0.916939000	-1.191862000	4.436728000
	H	-0.234069000	-2.059459000	3.045179000
	N	-0.602128000	1.265981000	3.331935000
	H	-0.573713000	1.390024000	4.436728000
	H	-1.666509000	1.232440000	3.045179000
	N	-2.428421000	-0.636041000	-2.696434000
	N	-2.486005000	-0.677072000	-1.358930000
	N	-3.333695000	-1.347195000	-3.500392000
	N	-3.432993000	-1.409985000	-0.754382000
	N	-4.324640000	-2.111491000	-2.880125000
	N	-4.382547000	-2.145986000	-1.487215000
	H	-3.261712000	-1.301986000	-4.589253000
	H	-5.039169000	-2.682745000	-3.476800000
	H	-5.133612000	-2.754530000	-0.981272000
	N	-3.433268000	-1.417787000	0.743704000
	N	-2.250565000	-1.243331000	1.357186000
	N	-4.618305000	-1.606753000	1.477125000
	N	-2.174982000	-1.255726000	2.696391000
	N	-4.553337000	-1.606462000	2.869936000
	N	-3.316136000	-1.431176000	3.493896000
	H	-5.576205000	-1.724714000	0.968702000
	H	-5.460352000	-1.735101000	3.464667000
	H	-3.233173000	-1.430090000	4.582734000
	N	0.663383000	2.421095000	-2.696434000
	N	0.656641000	2.491479000	-1.358930000
	N	0.500143000	3.560662000	-3.500392000

	N 0.495414000 3.678052000 -0.754382000<br>N 0.333715000 4.800994000 -2.880125000<br>N 0.332794000 4.868390000 -1.487215000<br>H 0.503303000 3.475718000 -4.589253000<br>H 0.196259000 5.705421000 -3.476800000<br>H 0.181313000 5.823103000 -0.981272000<br>N 0.488795000 3.682191000 0.743704000<br>N 0.048526000 2.570712000 1.357186000<br>N 0.917663000 4.802946000 1.477125000<br>N 0.000000000 2.511453000 2.696391000<br>N 0.885432000 4.746537000 2.869936000<br>N 0.418633000 3.587446000 3.493896000<br>H 1.294456000 5.691493000 0.968702000<br>H 1.227534000 5.596354000 3.464667000<br>H 0.378093000 3.515055000 4.582734000<br>N 1.765038000 -1.785054000 -2.696434000<br>N 1.829364000 -1.814407000 -1.358930000<br>N 2.833552000 -2.213467000 -3.500392000<br>N 2.937579000 -2.268067000 -0.754382000<br>N 3.990925000 -2.689502000 -2.880125000<br>N 4.049752000 -2.722403000 -1.487215000<br>H 2.758409000 -2.173732000 -4.589253000<br>H 4.842910000 -3.022676000 -3.476800000<br>H 4.952299000 -3.068573000 -0.981272000<br>N 2.944474000 -2.264404000 0.743704000<br>N 2.202039000 -1.327381000 1.357186000<br>N 3.700642000 -3.196193000 1.477125000<br>N 2.174982000 -1.255726000 2.696391000<br>N 3.667906000 -3.140075000 2.869936000<br>N 2.897503000 -2.156270000 3.493896000<br>H 4.281749000 -3.966778000 0.968702000<br>H 4.232818000 -3.861253000 3.464667000<br>H 2.855081000 -2.084965000 4.582734000<br>H -0.050527000 0.974013000 0.391751000<br>H 0.868784000 -0.443249000 0.391751000<br>H -0.818257000 -0.530764000 0.391751000<br>H 0.000000000 0.000000000 -1.000497000<br>N 0.000000000 0.000000000 0.035928000
H_3O	TPSSh/def2-SVP (0 imaginary Frequencies)
	O -0.000020000 0.000000000 0.109357000<br>H 0.972936000 0.000000000 -0.239783000<br>H -0.486460000 -0.842716000 -0.239744000<br>H -0.486460000 0.842716000 -0.239744000
H_3O^+	TPSSh/def2-SVP (0 imaginary Frequencies)
	O 0.000021000 0.000000000 0.060830000<br>H 0.945015000 0.000000000 -0.223633000<br>H -0.472519000 -0.818392000 -0.223555000

	H -0.472519000 0.818392000 -0.223555000
[H_3O⊂spherical cryptand]	TPSSh/def2-SVP (0 imaginary Frequencies)
	N -1.008949000 2.787176000 -0.116190000<br>N -1.081175000 -1.284983000 2.442283000<br>N 3.308473000 -0.032775000 0.033319000<br>N 3.641850000 0.082031000 -1.371602000<br>N 3.198127000 -1.096578000 -2.230610000<br>O 1.825049000 -1.341404000 -2.033999000<br>N 1.293414000 -2.327950000 -2.885797000<br>N -0.142841000 -2.640594000 -2.483957000<br>N -1.030099000 -1.469110000 -2.356705000<br>N -2.429630000 -1.862572000 -2.088403000<br>N -2.601708000 -2.901103000 -0.995832000<br>O -1.690093000 -2.622411000 0.051547000<br>N -1.960101000 -3.304380000 1.253835000<br>N -1.033813000 -2.751435000 2.319171000<br>N -0.948435000 -0.623880000 -3.559703000<br>N -1.872409000 0.577652000 -3.525100000<br>O -1.630767000 1.273234000 -2.324717000<br>N -2.540916000 2.327265000 -2.067693000<br>N -2.397859000 2.759184000 -0.620338000<br>N -0.097132000 3.474967000 -1.048973000<br>N 1.330272000 3.655836000 -0.547297000<br>O 1.842657000 2.421211000 -0.106081000<br>N 3.207188000 2.460514000 0.240766000<br>N 3.626514000 1.124792000 0.843945000<br>N -2.471674000 -0.821280000 2.634278000<br>N -2.611116000 0.648920000 2.981729000<br>O -1.674330000 1.392128000 2.223046000<br>N -1.901275000 2.782012000 2.221006000<br>N -0.950599000 3.406060000 1.218162000<br>N 3.603180000 -1.315308000 0.638614000<br>N 3.145275000 -1.458422000 2.085567000<br>O 1.779192000 -1.131554000 2.190001000<br>N 1.233560000 -1.361794000 3.467032000<br>N -0.191790000 -0.826131000 3.525771000<br>H 4.693992000 -1.567478000 0.627674000<br>H 3.080400000 -2.086849000 0.054058000<br>H 3.325651000 -2.509018000 2.395413000<br>H 3.745837000 -0.821849000 2.768527000<br>H 1.277158000 -2.442611000 3.715434000<br>H 1.819642000 -0.836002000 4.249823000<br>H -0.159010000 0.269806000 3.470804000<br>H -0.604757000 -1.108357000 4.519453000<br>H -3.011437000 -0.968357000 1.668629000<br>H -2.976279000 -1.415785000 3.432396000<br>H -3.642628000 0.964003000 2.691727000<br>H -2.467791000 0.847109000 4.063803000

	H -2.962128000 2.987033000 1.969850000<br>H -1.705912000 3.220985000 3.222208000<br>H 0.075960000 3.292734000 1.594494000<br>H -1.179427000 4.495415000 1.153995000<br>H -1.294939000 -3.232524000 3.290523000<br>H -0.002445000 -3.032498000 2.063348000<br>H -1.773830000 -4.393956000 1.147071000<br>H -3.028001000 -3.174915000 1.524768000<br>H -2.453936000 -3.937006000 -1.364205000<br>H -3.643055000 -2.803634000 -0.604220000<br>H -2.974509000 -0.951541000 -1.744125000<br>H -2.911747000 -2.251769000 -3.016417000<br>H -2.960953000 2.011685000 -0.012915000<br>H -2.876743000 3.760806000 -0.507914000<br>H -3.582311000 1.946907000 -2.201241000<br>H -2.370584000 3.160009000 -2.780658000<br>H -2.940183000 0.281614000 -3.577076000<br>H -1.661458000 1.217511000 -4.407927000<br>H -1.187252000 -1.219693000 -4.471292000<br>H 0.086681000 -0.264295000 -3.645764000<br>H -0.483220000 4.487190000 -1.302270000<br>H -0.073950000 2.880241000 -1.971501000<br>H 1.387710000 4.409275000 0.265628000<br>H 1.931674000 4.057010000 -1.389988000<br>H 3.400716000 3.249457000 0.997355000<br>H 3.822010000 2.718730000 -0.646781000<br>H 3.092842000 1.017900000 1.800060000<br>H 4.718072000 1.213451000 1.076766000<br>H 3.135520000 0.977474000 -1.761506000<br>H 4.738877000 0.209935000 -1.555320000<br>H 3.787180000 -2.010248000 -2.005753000<br>H 3.407008000 -0.840826000 -3.290439000<br>H 1.873307000 -3.271758000 -2.810699000<br>H 1.362201000 -2.000890000 -3.944131000<br>H -0.542471000 -3.356009000 -3.236317000<br>H -0.134664000 -3.143827000 -1.508269000<br>H -0.658150000 -0.483029000 0.850073000<br>H -0.640218000 -0.498818000 -0.855112000<br>H -0.633798000 0.983692000 -0.015659000<br>O -0.365572000 -0.001881000 -0.003891000
[H_3O^+⊂spherical cryptand]	TPSSh/def2-SVP (0 imaginary Frequencies)
	N -1.038816000 2.291278000 1.545763000<br>N -1.036347000 -2.482908000 1.216224000<br>N 3.337827000 0.002049000 -0.009351000<br>N 3.664967000 0.953579000 -1.054601000<br>N 3.202758000 0.546473000 -2.447328000<br>O 1.823722000 0.227586000 -2.419114000

	N	1.296767000	-0.104762000	-3.679421000
	N	-0.110566000	-0.660865000	-3.516644000
	N	-1.050892000	0.189647000	-2.753355000
	N	-2.435357000	-0.305030000	-2.884833000
	N	-2.576539000	-1.791075000	-2.590513000
	O	-1.906072000	-2.052016000	-1.376540000
	N	-1.942740000	-3.393130000	-0.936748000
	N	-0.931260000	-3.537389000	0.190218000
	N	-0.945102000	1.605058000	-3.154544000
	N	-1.951176000	2.511041000	-2.460968000
	O	-1.907952000	2.221249000	-1.079835000
	N	-2.572291000	3.143274000	-0.243235000
	N	-2.424515000	2.654640000	1.190106000
	N	-0.098469000	3.376155000	1.186373000
	N	1.311317000	3.237292000	1.742780000
	O	1.834677000	1.978674000	1.398530000
	N	3.214924000	1.841439000	1.681840000
	N	3.672954000	0.430990000	1.335506000
	N	-2.419403000	-2.354021000	1.715395000
	N	-2.560041000	-1.356738000	2.855802000
	O	-1.895074000	-0.172598000	2.472481000
	N	-1.932041000	0.878604000	3.414262000
	N	-0.926441000	1.930649000	2.971555000
	N	3.669806000	-1.377510000	-0.311938000
	N	3.215369000	-2.381902000	0.738630000
	O	1.836739000	-2.202809000	1.005912000
	N	1.316122000	-3.130421000	1.925200000
	N	-0.091176000	-2.716391000	2.330811000
	H	4.764549000	-1.546638000	-0.458455000
	H	3.169204000	-1.642192000	-1.255086000
	H	3.411235000	-3.400818000	0.348489000
	H	3.796449000	-2.284314000	1.677270000
	H	1.333101000	-4.155815000	1.502857000
	H	1.927164000	-3.163000000	2.850078000
	H	-0.026374000	-1.779438000	2.901627000
	H	-0.477012000	-3.506430000	3.009010000
	H	-3.039967000	-2.011537000	0.875084000
	H	-2.818124000	-3.330530000	2.062567000
	H	-3.636942000	-1.174319000	3.038387000
	H	-2.132383000	-1.747438000	3.799246000
	H	-2.957627000	1.288182000	3.496962000
	H	-1.644178000	0.520709000	4.421661000
	H	0.086009000	1.529954000	3.119211000
	H	-1.048674000	2.818782000	3.626454000
	H	-1.052858000	-4.548424000	0.632528000
	H	0.079055000	-3.466743000	-0.236037000
	H	-1.660345000	-4.087126000	-1.751874000
	H	-2.967065000	-3.667944000	-0.617914000

	H -2.153441000 -2.413289000 -3.402587000<br>H -3.653431000 -2.039072000 -2.518811000<br>H -3.051957000 0.252487000 -2.165473000<br>H -2.838387000 -0.117099000 -3.902339000<br>H -3.041818000 1.753765000 1.316170000<br>H -2.822950000 3.442278000 1.863862000<br>H -3.650340000 3.207115000 -0.488294000<br>H -2.148061000 4.156912000 -0.378078000<br>H -2.977790000 2.374410000 -2.853131000<br>H -1.667577000 3.563375000 -2.655839000<br>H -1.071986000 1.727552000 -4.250689000<br>H 0.067232000 1.936964000 -2.885393000<br>H -0.486332000 4.357588000 1.531778000<br>H -0.037627000 3.400944000 0.089352000<br>H 1.331965000 3.385719000 2.841698000<br>H 1.918073000 4.055971000 1.305232000<br>H 3.414405000 2.013723000 2.758540000<br>H 3.791176000 2.607543000 1.125737000<br>H 3.177186000 -0.255332000 2.037951000<br>H 4.768597000 0.392475000 1.551413000<br>H 3.166516000 1.903141000 -0.809509000<br>H 4.759488000 1.164048000 -1.133688000<br>H 3.780617000 -0.316151000 -2.834847000<br>H 3.396525000 1.393376000 -3.135788000<br>H 1.904326000 -0.890696000 -4.172396000<br>H 1.311879000 0.773187000 -4.356949000<br>H -0.501414000 -0.853400000 -4.537993000<br>H -0.044813000 -1.623326000 -2.990077000<br>H -0.674937000 -0.892754000 0.433889000<br>H -0.681138000 0.072141000 -0.986822000<br>H -0.676192000 0.820105000 0.559218000<br>O -0.421365000 0.000058000 0.001140000
[H_3O⊂[2.2.2]cryptand]	TPSSh/def2-SVP (0 imaginary Frequencies)
	O -1.396271000 -2.354366000 -0.167632000<br>O 1.523566000 -2.506229000 -0.271539000<br>O 1.524406000 1.486531000 -2.034474000<br>O -1.394817000 1.325789000 -1.952158000<br>O -1.391146000 1.034085000 2.123176000<br>O 1.529068000 1.018445000 2.302972000<br>N 3.263520000 -0.001548000 -0.001528000<br>N -3.167564000 0.000882000 0.002263000<br>N -2.775527000 0.720731000 2.379711000<br>H -3.137531000 1.462440000 3.130257000<br>H -2.847423000 -0.288197000 2.827803000<br>N -3.596593000 0.858636000 1.116649000<br>H -4.675887000 0.674770000 1.356672000<br>H -3.521052000 1.907010000 0.790622000

	N	-2.782615000	-2.419660000	-0.562625000
	H	-3.143694000	-3.439873000	-0.291115000
	H	-2.857686000	-2.306342000	-1.660553000
	N	-3.600482000	-1.392268000	0.187943000
	H	-4.680183000	-1.504423000	-0.091790000
	H	-3.526299000	-1.634057000	1.258978000
	N	-2.781094000	1.700757000	-1.811451000
	H	-3.141954000	1.975123000	-2.831062000
	H	-2.856144000	2.595323000	-1.164970000
	N	-3.599131000	0.537161000	-1.297173000
	H	-4.678951000	0.835211000	-1.255705000
	H	-3.523652000	-0.269520000	-2.042018000
	N	-0.625740000	1.283322000	3.312561000
	H	-0.457357000	0.339725000	3.863759000
	H	-1.223198000	1.973948000	3.949734000
	N	0.685388000	1.936800000	2.961779000
	H	0.487203000	2.822652000	2.325245000
	H	1.169803000	2.299922000	3.899728000
	N	-0.632600000	-3.510677000	-0.546387000
	H	-0.465366000	-3.516977000	-1.639306000
	H	-1.231880000	-4.406328000	-0.265425000
	N	0.678803000	-3.535315000	0.194147000
	H	0.481139000	-3.427354000	1.279682000
	H	1.162427000	-4.529530000	0.039014000
	N	-0.631039000	2.230157000	-2.766095000
	H	-0.462473000	3.180387000	-2.226594000
	H	-1.231069000	2.433901000	-3.681939000
	N	0.679290000	1.599327000	-3.158181000
	H	0.480047000	0.605581000	-3.607503000
	H	1.163576000	2.230237000	-3.941673000
	N	2.721822000	-2.399315000	0.473148000
	H	3.230677000	-3.386241000	0.508986000
	H	2.497366000	-2.103435000	1.517598000
	N	3.651477000	-1.393149000	-0.191311000
	H	4.691648000	-1.582883000	0.163889000
	H	3.646250000	-1.618935000	-1.268581000
	N	2.728730000	1.608461000	1.839826000
	H	3.239046000	2.128821000	2.678317000
	H	2.506381000	2.368604000	1.064177000
	N	3.655850000	0.529528000	1.297290000
	H	4.696092000	0.931773000	1.280923000
	H	3.652788000	-0.290779000	2.031223000
	N	2.721660000	0.786924000	-2.315228000
	H	3.230635000	1.249249000	-3.187833000
	H	2.495871000	-0.265099000	-2.581919000
	N	3.652377000	0.857439000	-1.112336000
	H	4.691738000	0.642236000	-1.455252000
	H	3.649902000	1.903376000	-0.769459000

	H	-0.677254000	-0.933056000	-0.105754000
	H	-0.676050000	0.556172000	-0.756746000
	H	-0.672735000	0.375133000	0.857110000
	O	-0.277295000	-0.001389000	-0.002503000
[H_3O^+⊂[2.2.2]cryptand]	TPSSh/def2-SVP (0 imaginary Frequencies)			
	O	1.398132000	-2.336624000	-0.374054000
	O	-1.489786000	-2.475094000	-0.353198000
	O	-1.489817000	0.931476000	2.319770000
	O	1.397872000	0.844055000	2.210847000
	O	1.398555000	1.492696000	-1.836361000
	O	-1.488974000	1.543411000	-1.966356000
	N	-3.224335000	-0.000038000	-0.000219000
	N	3.195371000	-0.000155000	0.000182000
	N	2.700724000	1.038755000	-2.219086000
	H	3.126732000	1.750000000	-2.947734000
	H	2.623461000	0.055758000	-2.719737000
	N	3.605544000	0.991212000	-0.997906000
	H	4.649275000	0.827711000	-1.340861000
	H	3.579973000	1.988036000	-0.532866000
	N	2.700362000	-2.441488000	0.210211000
	H	3.126107000	-3.428197000	-0.041641000
	H	2.623297000	-2.383746000	1.311866000
	N	3.605362000	-1.360284000	-0.359220000
	H	4.649038000	-1.575678000	-0.046060000
	H	3.579838000	-1.456039000	-1.455013000
	N	2.700202000	1.402289000	2.009555000
	H	3.125960000	1.677343000	2.990044000
	H	2.623320000	2.327569000	1.408835000
	N	3.605120000	0.368502000	1.357887000
	H	4.648848000	0.747246000	1.388108000
	H	3.579371000	-0.532676000	1.988586000
	N	0.627381000	2.006304000	-2.933229000
	H	0.416351000	1.199570000	-3.657556000
	H	1.214211000	2.792755000	-3.438653000
	N	-0.666556000	2.590072000	-2.416998000
	H	-0.448722000	3.313894000	-1.605143000
	H	-1.147811000	3.154322000	-3.242121000
	N	0.626870000	-3.543332000	-0.270780000
	H	0.415668000	-3.767452000	0.789959000
	H	1.213758000	-4.374200000	-0.699206000
	N	-0.666955000	-3.388069000	-1.034636000
	H	-0.448983000	-3.046246000	-2.067168000
	H	-1.147906000	-4.384868000	-1.111403000
	N	0.626604000	1.537291000	3.203932000
	H	0.415564000	2.567874000	2.867243000
	H	1.213378000	1.581955000	4.137757000
	N	-0.667343000	0.798390000	3.451477000

	H -0.449561000 -0.266616000 3.672398000<br>H -1.148503000 1.230893000 4.352720000<br>N -2.719988000 -2.230587000 -1.017444000<br>H -3.223367000 -3.193219000 -1.237045000<br>H -2.534591000 -1.725344000 -1.985417000<br>N -3.628640000 -1.396846000 -0.126491000<br>H -4.671495000 -1.494767000 -0.502766000<br>H -3.614244000 -1.862584000 0.870601000<br>N -2.719605000 1.996248000 -1.423327000<br>H -3.222736000 2.667345000 -2.147739000<br>H -2.534889000 2.582368000 -0.501942000<br>N -3.628226000 0.807717000 -1.146926000<br>H -4.671127000 1.182521000 -1.043951000<br>H -3.613535000 0.177023000 -2.048769000<br>N -2.720289000 0.234461000 2.440227000<br>H -3.223684000 0.526145000 3.383517000<br>H -2.535352000 -0.856502000 2.487121000<br>N -3.628690000 0.589173000 1.272508000<br>H -4.671648000 0.312529000 1.545333000<br>H -3.614058000 1.685540000 1.177217000<br>H 0.635237000 -0.933685000 -0.133751000<br>H 0.634794000 0.351393000 0.875626000<br>H 0.635441000 0.583036000 -0.742034000<br>O 0.259527000 0.000267000 -0.000226000
[H_3O⊂[bpy.bpy.bpy]cryptand]	TPSSh/def2-SVP (0 imaginary Frequencies)
	N -2.880991000 -0.043392000 0.071816000<br>N 2.872131000 0.050297000 -0.077597000<br>N 3.233133000 1.252373000 0.647032000<br>H 4.337958000 1.411138000 0.687956000<br>H 2.893049000 1.122980000 1.688078000<br>N 3.190285000 0.094825000 -1.491140000<br>H 4.290562000 0.115998000 -1.682291000<br>H 2.781022000 1.037227000 -1.891700000<br>N 3.276880000 -1.176830000 0.579546000<br>H 4.386930000 -1.288159000 0.632422000<br>H 2.903158000 -2.015617000 -0.031076000<br>N -3.244774000 -1.435027000 -0.130799000<br>H -4.348058000 -1.582532000 -0.168289000<br>H -2.879789000 -2.001429000 0.742942000<br>N -3.305949000 0.830163000 -1.008342000<br>H -4.413815000 0.934278000 -1.056733000<br>H -2.994250000 0.359900000 -1.956725000<br>N -3.224643000 0.460775000 1.390822000<br>H -4.323954000 0.463104000 1.568667000<br>H -2.894828000 1.512768000 1.437034000<br>N 2.596833000 2.526066000 0.111697000<br>N 1.274803000 2.530537000 -0.080495000<br>N 3.392944000 3.659591000 -0.125425000

	N	0.662912000	3.645166000	-0.529680000
	N	2.768926000	4.830119000	-0.577153000
	N	1.395285000	4.829575000	-0.786764000
	H	4.472731000	3.620211000	0.033880000
	H	3.355608000	5.729928000	-0.778866000
	H	0.905823000	5.727287000	-1.166185000
	N	-0.796205000	3.563594000	-0.754148000
	N	-1.382636000	2.333028000	-0.722032000
	N	-1.569940000	4.719840000	-1.006139000
	N	-2.701226000	2.223212000	-0.957207000
	N	-2.933776000	4.605943000	-1.231473000
	N	-3.519969000	3.330146000	-1.209928000
	H	-1.102045000	5.704341000	-1.005196000
	H	-3.540884000	5.495748000	-1.413940000
	H	-4.589425000	3.193471000	-1.381199000
	N	2.718631000	-1.348533000	1.983728000
	N	1.402548000	-1.192189000	2.151340000
	N	3.574484000	-1.698594000	3.041275000
	N	0.854437000	-1.359684000	3.372845000
	N	3.017138000	-1.891305000	4.313406000
	N	1.649918000	-1.718921000	4.488701000
	H	4.647671000	-1.809684000	2.871457000
	H	3.651245000	-2.157359000	5.162946000
	H	1.212467000	-1.836745000	5.480876000
	N	-0.599821000	-1.132491000	3.489094000
	N	-1.242572000	-0.537819000	2.441705000
	N	-1.315472000	-1.504448000	4.650926000
	N	-2.559959000	-0.285366000	2.534488000
	N	-2.677064000	-1.257666000	4.736740000
	N	-3.321109000	-0.629914000	3.656559000
	H	-0.802795000	-2.003911000	5.473215000
	H	-3.239024000	-1.554381000	5.625419000
	H	-4.391124000	-0.414848000	3.680234000
	N	2.594299000	-1.045957000	-2.301516000
	N	1.283933000	-1.275348000	-2.179749000
	N	3.408394000	-1.793221000	-3.169691000
	N	0.702205000	-2.258542000	-2.894927000
	N	2.815181000	-2.810224000	-3.929658000
	N	1.453747000	-3.053698000	-3.793817000
	H	4.478472000	-1.586228000	-3.239976000
	H	3.417252000	-3.418160000	-4.609717000
	H	0.989342000	-3.863442000	-4.357956000
	N	-0.745098000	-2.473520000	-2.680657000
	N	-1.332418000	-1.861867000	-1.612854000
	N	-1.503875000	-3.295404000	-3.544113000
	N	-2.640361000	-2.060595000	-1.376206000
	N	-2.856937000	-3.488603000	-3.305731000

	N -3.445025000 -2.860130000 -2.196373000<br>H -1.034244000 -3.761711000 -4.410387000<br>H -3.454210000 -4.111053000 -3.976092000<br>H -4.506042000 -2.982966000 -1.970599000<br>H -0.432416000 -0.216369000 0.948354000<br>H -0.463964000 -0.708901000 -0.631313000<br>H -0.482739000 0.904928000 -0.268974000<br>O -0.107481000 0.000147000 0.005370000
[H_3O^+⊂[bpy.bpy.bpy]cryptand]	TPSSh/def2-SVP (0 imaginary Frequencies)
	N -2.914689000 -0.001057000 -0.000671000<br>N 2.909946000 0.001865000 0.001166000<br>N 3.285904000 0.575559000 -1.280567000<br>H 4.390466000 0.612449000 -1.424713000<br>H 2.927176000 1.617701000 -1.296894000<br>N 3.286948000 -1.394608000 0.145568000<br>H 4.391588000 -1.536921000 0.186956000<br>H 2.929758000 -1.930043000 -0.749280000<br>N 3.284894000 0.825507000 1.138866000<br>H 4.389340000 0.932957000 1.243027000<br>H 2.926505000 0.318229000 2.049498000<br>N -3.310630000 -0.186144000 1.388693000<br>H -4.413254000 -0.266931000 1.505123000<br>H -3.000936000 0.713966000 1.946528000<br>N -3.310311000 -1.112006000 -0.855383000<br>H -4.412895000 -1.172672000 -0.983752000<br>H -3.000565000 -2.044922000 -0.354377000<br>N -3.311910000 1.294256000 -0.535082000<br>H -4.414645000 1.434479000 -0.522898000<br>H -3.002613000 1.327488000 -1.593636000<br>N 2.674039000 -0.141268000 -2.470966000<br>N 1.340178000 -0.255449000 -2.509593000<br>N 3.491361000 -0.640062000 -3.498954000<br>N 0.761372000 -0.875106000 -3.545527000<br>N 2.894813000 -1.283071000 -4.584491000<br>N 1.507220000 -1.412214000 -4.612058000<br>H 4.576487000 -0.532209000 -3.439188000<br>H 3.504390000 -1.693688000 -5.392365000<br>H 1.022833000 -1.942788000 -5.432583000<br>N -0.727468000 -0.995672000 -3.510037000<br>N -1.336254000 -0.924460000 -2.307649000<br>N -1.463120000 -1.197965000 -4.691803000<br>N -2.669369000 -1.090598000 -2.230739000<br>N -2.845200000 -1.347371000 -4.618873000<br>N -3.460618000 -1.302229000 -3.367709000<br>H -0.959367000 -1.215397000 -5.658211000<br>H -3.435924000 -1.492349000 -5.525893000<br>H -4.540818000 -1.423644000 -3.266766000<br>N 2.671595000 2.214192000 1.113053000

	N 1.337607000 2.303312000 1.034038000<br>N 3.487738000 3.354740000 1.194375000<br>N 0.757496000 3.509668000 1.015286000<br>N 2.889844000 4.615702000 1.180308000<br>N 1.502099000 4.702679000 1.082887000<br>H 4.573006000 3.250235000 1.257383000<br>H 3.498491000 5.521302000 1.228175000<br>H 1.016649000 5.678053000 1.033729000<br>N -0.731406000 3.537617000 0.893389000<br>N -1.338979000 2.460251000 0.353474000<br>N -1.468268000 4.661190000 1.309585000<br>N -2.672148000 2.475255000 0.171210000<br>N -2.850358000 4.671296000 1.143744000<br>N -3.464573000 3.564721000 0.556841000<br>H -0.965403000 5.507150000 1.778072000<br>H -3.442014000 5.528500000 1.472073000<br>H -4.544752000 3.536871000 0.401253000<br>N 2.674171000 -2.068021000 1.360599000<br>N 1.340201000 -2.045036000 1.477532000<br>N 3.490850000 -2.709435000 2.306756000<br>N 0.760583000 -2.633924000 2.530857000<br>N 2.893479000 -3.329683000 3.404990000<br>N 1.505721000 -3.289975000 3.529158000<br>H 4.576108000 -2.710810000 2.184635000<br>H 3.502532000 -3.824571000 4.164590000<br>H 1.020662000 -3.736754000 4.397789000<br>N -0.728370000 -2.543495000 2.616135000<br>N -1.336697000 -1.537371000 1.953522000<br>N -1.464560000 -3.466733000 3.380549000<br>N -2.669997000 -1.388231000 2.057593000<br>N -2.846784000 -3.329434000 3.472026000<br>N -3.461791000 -2.268051000 2.807594000<br>H -0.961046000 -4.295202000 3.878664000<br>H -3.437918000 -4.043173000 4.049774000<br>H -4.542103000 -2.120332000 2.861156000<br>H -0.471760000 0.945579000 0.127358000<br>H -0.470079000 -0.582445000 0.755307000<br>H -0.470826000 -0.362239000 -0.881881000<br>O -0.118467000 0.000656000 0.000171000
[K⊂[2.2.2]cryptand]	TPSSh/def2-SVP (0 imaginary Frequencies)
	O 1.415623000 -1.259702000 -2.111810000<br>O -1.413822000 -1.475687000 -1.966996000<br>O -1.415283000 -0.965483000 2.261226000<br>O 1.414237000 -1.199162000 2.147011000<br>O 1.414510000 2.459163000 -0.034600000<br>O -1.414897000 2.441269000 -0.295283000<br>N -3.087285000 -0.000286000 -0.000455000

	N	3.087038000	0.000118000	0.000425000
	N	2.771101000	2.456782000	-0.445050000
	H	3.240854000	3.429621000	-0.196738000
	H	2.837446000	2.338384000	-1.544368000
	N	3.529127000	1.367052000	0.294922000
	H	4.614695000	1.496912000	0.084708000
	H	3.390626000	1.551174000	1.370153000
	N	2.772201000	-1.613973000	-1.904477000
	H	3.242121000	-1.884990000	-2.871163000
	H	2.838551000	-2.507063000	-1.252609000
	N	3.530141000	-0.428444000	-1.330218000
	H	4.615636000	-0.675772000	-1.336614000
	H	3.392591000	0.410736000	-2.027403000
	N	2.770737000	-0.842439000	2.350636000
	H	3.240358000	-1.544026000	3.068952000
	H	2.836935000	0.168696000	2.798030000
	N	3.529184000	-0.938133000	1.037157000
	H	4.614635000	-0.820531000	1.255070000
	H	3.391263000	-1.961451000	0.658996000
	N	0.687970000	3.561187000	-0.551796000
	H	0.605665000	3.488150000	-1.653586000
	H	1.208498000	4.509870000	-0.309343000
	N	-0.688553000	3.602931000	0.069089000
	H	-0.606199000	3.678596000	1.170702000
	H	-1.209281000	4.510332000	-0.298619000
	N	0.689107000	-2.258778000	-2.807377000
	H	0.606338000	-3.176143000	-2.192825000
	H	1.209929000	-2.523734000	-3.749870000
	N	-0.687144000	-1.741830000	-3.154813000
	H	-0.604348000	-0.826066000	-3.771731000
	H	-1.207818000	-2.514255000	-3.756472000
	N	0.687510000	-1.302536000	3.359846000
	H	0.604976000	-0.311902000	3.847594000
	H	1.208083000	-1.986818000	4.060210000
	N	-0.688854000	-1.861439000	3.085457000
	H	-0.606304000	-2.853470000	2.600578000
	H	-1.209670000	-1.996436000	4.055133000
	N	-2.770472000	-1.150036000	-2.216590000
	H	-3.239777000	-1.941814000	-2.834311000
	H	-2.837066000	-0.208054000	-2.795540000
	N	-3.528976000	-1.069124000	-0.902115000
	H	-4.614453000	-0.982235000	-1.133889000
	H	-3.390684000	-2.032491000	-0.390250000
	N	-2.771499000	2.493902000	0.111745000
	H	-3.241291000	3.424624000	-0.264813000
	H	-2.837844000	2.524027000	1.217015000
	N	-3.529581000	1.314773000	-0.475360000
	H	-4.615105000	1.471599000	-0.284117000

	H -3.391501000 1.353133000 -1.565623000<br>N -2.771882000 -1.344384000 2.103696000<br>H -3.241555000 -1.483093000 3.098141000<br>H -2.838411000 -2.316929000 1.577706000<br>N -3.529980000 -0.246663000 1.375710000<br>H -4.615460000 -0.490951000 1.415557000<br>H -3.392219000 0.678417000 1.954025000<br>K -0.000118000 0.000466000 -0.000086000
[K$^+$⊂[2.2.2]cryptand]	TPSSh/def2-SVP (0 imaginary Frequencies)
	O 1.419125000 2.462646000 0.227381000<br>O -1.419370000 2.473218000 -0.012869000<br>O -1.419502000 -1.248190000 -2.134623000<br>O 1.419566000 -1.034469000 -2.245567000<br>O 1.419378000 -1.428542000 2.018192000<br>O -1.419641000 -1.225723000 2.147291000<br>N -3.089229000 0.000265000 -0.000088000<br>N 3.089245000 0.000332000 0.000188000<br>N 2.765483000 -1.050732000 2.264131000<br>H 3.247702000 -1.789691000 2.932629000<br>H 2.797336000 -0.074066000 2.785125000<br>N 3.537218000 -1.021756000 0.954655000<br>H 4.617772000 -0.910118000 1.191233000<br>H 3.415995000 -2.009360000 0.486165000<br>N 2.765359000 2.486406000 -0.222256000<br>H 3.247633000 3.434897000 0.083154000<br>H 2.797426000 2.448558000 -1.328528000<br>N 3.536759000 1.338097000 0.408153000<br>H 4.617459000 1.487270000 0.193917000<br>H 3.414743000 1.426307000 1.497576000<br>N 2.766109000 -1.435186000 -2.041904000<br>H 3.248145000 -1.644070000 -3.016331000<br>H 2.799222000 -2.374753000 -1.456719000<br>N 3.537050000 -0.314963000 -1.362291000<br>H 4.617852000 -0.574644000 -1.384297000<br>H 3.414772000 0.584456000 -1.983229000<br>N 0.681956000 -1.658889000 3.207990000<br>H 0.574426000 -0.720279000 3.785618000<br>H 1.205561000 -2.395097000 3.847736000<br>N -0.682415000 -2.207049000 2.858559000<br>H -0.574963000 -3.127060000 2.251724000<br>H -1.206102000 -2.475984000 3.796030000<br>N 0.682094000 3.608310000 -0.168357000<br>H 0.574683000 3.639015000 -1.270047000<br>H 1.205895000 4.530390000 0.149206000<br>N -0.682261000 3.580051000 0.481029000<br>H -0.574804000 3.514947000 1.581220000<br>H -1.205987000 4.526293000 0.244842000

	N 0.682377000 -1.950551000 -3.039157000<br>H 0.574947000 -2.919666000 -2.514291000<br>H 1.206104000 -2.137227000 -3.996393000<br>N -0.681979000 -1.374209000 -3.339822000<br>H -0.574450000 -0.388937000 -3.833669000<br>H -1.205454000 -2.052076000 -4.041191000<br>N -2.765506000 2.457642000 0.437362000<br>H -3.247951000 3.429016000 0.215569000<br>H -2.797598000 2.323911000 1.536179000<br>N -3.536752000 1.368376000 -0.290424000<br>H -4.617503000 1.498289000 -0.064239000<br>H -3.414511000 1.550838000 -1.368062000<br>N -2.766019000 -1.607482000 1.909165000<br>H -3.248204000 -1.900845000 2.861496000<br>H -2.798684000 -2.492299000 1.244058000<br>N -3.537209000 -0.432434000 1.329719000<br>H -4.617955000 -0.693292000 1.328777000<br>H -3.415310000 0.409532000 2.026672000<br>N -2.765293000 -0.849580000 -2.346834000<br>H -3.247808000 -1.526929000 -3.077510000<br>H -2.796524000 0.168979000 -2.780270000<br>N -3.537219000 -0.934820000 -1.039961000<br>H -4.617747000 -0.802805000 -1.265972000<br>H -3.416240000 -1.959511000 -0.659341000<br>K 0.000267000 -0.000372000 0.000183000
[Na⊂[bpy.bpy.bpy]cryptand]	TPSSh/def2-SVP (0 imaginary Frequencies)
	Na 0.001373000 -0.003423000 -0.016846000<br>N -0.775668000 -2.602563000 0.549048000<br>N 0.778039000 2.607134000 -0.522588000<br>N 0.755919000 3.304150000 0.754779000<br>H 1.008462000 4.383776000 0.649747000<br>H 1.537600000 2.856142000 1.390827000<br>N -0.200534000 3.134639000 -1.463048000<br>H 0.041706000 4.173160000 -1.783791000<br>H -1.170686000 3.180064000 -0.940700000<br>N 2.114043000 2.534998000 -1.094485000<br>H 2.543916000 3.543965000 -1.288997000<br>H 2.028586000 2.032635000 -2.072589000<br>N -0.000837000 -3.450645000 -0.345799000<br>H -0.268814000 -4.526620000 -0.243303000<br>H 1.058989000 -3.356778000 -0.056040000<br>N -2.204270000 -2.677492000 0.284332000<br>H -2.627604000 -3.675809000 0.538964000<br>H -2.347635000 -2.540002000 -0.800563000<br>N -0.457098000 -2.830934000 1.950896000<br>H -0.691585000 -3.871332000 2.271737000<br>H -1.097857000 -2.161888000 2.549337000<br>N -0.570244000 3.180721000 1.480434000

	N	-1.219647000	2.018125000	1.367552000
	N	-1.052495000	4.254074000	2.245398000
	N	-2.409214000	1.856236000	1.996452000
	N	-2.262810000	4.085474000	2.931567000
	N	-2.949704000	2.885183000	2.803995000
	H	-0.493205000	5.190453000	2.300001000
	H	-2.667014000	4.888387000	3.553029000
	H	-3.891523000	2.742294000	3.334222000
	N	-3.108374000	0.570533000	1.800361000
	N	-2.429583000	-0.415457000	1.163228000
	N	-4.434050000	0.366630000	2.249436000
	N	-3.006815000	-1.610029000	1.002931000
	N	-5.035140000	-0.872397000	2.066449000
	N	-4.307170000	-1.896476000	1.445984000
	H	-4.991165000	1.172016000	2.728663000
	H	-6.058272000	-1.044946000	2.409404000
	H	-4.733162000	-2.892106000	1.304714000
	N	3.089418000	1.744730000	-0.243776000
	N	2.605893000	0.688421000	0.417232000
	N	4.439288000	2.124392000	-0.195670000
	N	3.439079000	-0.049000000	1.192434000
	N	5.318815000	1.344669000	0.567685000
	N	4.818537000	0.255122000	1.269197000
	H	4.786791000	3.003484000	-0.742597000
	H	6.381904000	1.592453000	0.618897000
	H	5.493450000	-0.359208000	1.865583000
	N	2.846736000	-1.175824000	1.940513000
	N	1.556343000	-1.489882000	1.670097000
	N	3.585728000	-1.906364000	2.901653000
	N	0.989149000	-2.528187000	2.292161000
	N	2.986765000	-2.974205000	3.556900000
	N	1.663535000	-3.311767000	3.241110000
	H	4.614344000	-1.634784000	3.139748000
	H	3.544622000	-3.546468000	4.302265000
	H	1.163429000	-4.158325000	3.716297000
	N	-0.367570000	2.267143000	-2.695355000
	N	-0.303013000	0.942381000	-2.518350000
	N	-0.597992000	2.863777000	-3.942271000
	N	-0.445142000	0.122015000	-3.591610000
	N	-0.784440000	2.025837000	-5.052563000
	N	-0.702894000	0.651132000	-4.879030000
	H	-0.630576000	3.951176000	-4.038150000
	H	-0.982091000	2.446466000	-6.041561000
	H	-0.843991000	-0.009151000	-5.735028000
	N	-0.322341000	-1.327438000	-3.354564000
	N	-0.202867000	-1.739308000	-2.065773000
	N	-0.325472000	-2.263068000	-4.416847000

	N	-0.130142000	-3.049104000	-1.802267000
	N	-0.233330000	-3.619483000	-4.137275000
	N	-0.149278000	-4.033989000	-2.799031000
	H	-0.393518000	-1.930124000	-5.452759000
	H	-0.235597000	-4.351538000	-4.948573000
	H	-0.099033000	-5.092023000	-2.533055000
[Na^+⊂[bpy.bpy.bpy]cryptand]	TPSSh/def2-SVP (0 imaginary Frequencies)			
	Na	0.002560000	0.001821000	-0.001976000
	N	-0.045042000	0.006785000	2.780658000
	N	0.046163000	-0.005812000	-2.780767000
	N	0.497148000	-1.330669000	-3.184290000
	H	0.639909000	-1.412258000	-4.284185000
	H	-0.296204000	-2.048880000	-2.917702000
	N	0.977238000	1.040151000	-3.181488000
	H	1.000078000	1.186526000	-4.283705000
	H	1.989799000	0.716081000	-2.888131000
	N	-1.315965000	0.270724000	-3.216420000
	H	-1.429246000	0.200805000	-4.320538000
	H	-1.548410000	1.313730000	-2.943550000
	N	-1.208271000	0.789160000	3.175375000
	H	-1.265855000	0.935622000	4.276363000
	H	-2.107687000	0.218870000	2.888673000
	N	1.204155000	0.619552000	3.211586000
	H	1.330617000	0.589992000	4.316115000
	H	1.168043000	1.685061000	2.929145000
	N	-0.149148000	-1.386032000	3.193978000
	H	-0.264999000	-1.493303000	4.294818000
	H	0.798305000	-1.884476000	2.929000000
	N	1.772097000	-1.772125000	-2.491286000
	N	2.009911000	-1.292900000	-1.264774000
	N	2.640795000	-2.673802000	-3.126866000
	N	3.128632000	-1.664580000	-0.616278000
	N	3.780404000	-3.096105000	-2.444646000
	N	4.035081000	-2.583483000	-1.172490000
	H	2.420258000	-3.035812000	-4.133391000
	H	4.468593000	-3.811774000	-2.899628000
	H	4.917660000	-2.906141000	-0.619853000
	N	3.360756000	-1.055560000	0.726926000
	N	2.293431000	-0.508720000	1.336900000
	N	4.634006000	-1.053625000	1.322076000
	N	2.432191000	0.010572000	2.562313000
	N	4.783580000	-0.497992000	2.592858000
	N	3.664399000	0.028499000	3.235346000
	H	5.498281000	-1.465049000	0.800319000
	H	5.762149000	-0.483522000	3.077607000
	H	3.738134000	0.450886000	4.239798000
	N	-2.353123000	-0.620945000	-2.560771000
	N	-2.090336000	-1.078382000	-1.331002000

	N -3.549563000 -0.917951000 -3.232936000<br>N -2.987533000 -1.869694000 -0.715337000<br>N -4.502010000 -1.702626000 -2.585036000<br>N -4.219784000 -2.192073000 -1.309666000<br>H -3.725518000 -0.535932000 -4.240840000<br>H -5.452258000 -1.938089000 -3.069160000<br>H -4.954190000 -2.802510000 -0.783911000<br>N -2.612882000 -2.388334000 0.633634000<br>N -1.620976000 -1.745227000 1.276077000<br>N -3.265661000 -3.495896000 1.201800000<br>N -1.274285000 -2.136899000 2.507853000<br>N -2.894289000 -3.915383000 2.479228000<br>N -1.894850000 -3.217428000 3.154995000<br>H -4.041088000 -4.032301000 0.654620000<br>H -3.385693000 -4.773195000 2.943424000<br>H -1.593586000 -3.502100000 4.165422000<br>N 0.708690000 2.376338000 -2.515888000<br>N 0.145102000 2.363239000 -1.302295000<br>N 1.073366000 3.568525000 -3.161900000<br>N -0.105764000 3.528742000 -0.678597000<br>N 0.855341000 4.777817000 -2.504155000<br>N 0.252858000 4.763507000 -1.246047000<br>H 1.521553000 3.541602000 -4.157483000<br>H 1.144293000 5.723669000 -2.967480000<br>H 0.079248000 5.698078000 -0.712231000<br>N -0.783025000 3.448158000 0.649348000<br>N -0.733698000 2.262486000 1.283502000<br>N -1.441101000 4.558071000 1.206290000<br>N -1.284188000 2.143906000 2.497270000<br>N -2.029805000 4.431254000 2.464651000<br>N -1.937958000 3.211685000 3.133034000<br>H -1.507026000 5.501713000 0.664326000<br>H -2.547873000 5.278101000 2.919900000<br>H -2.366526000 3.081549000 4.129121000
NH_4	PBE0/def2-SVP (0 imaginary Frequencies)
	N 0.000000000 0.000000000 0.000000000<br>H -0.602138000 0.602138000 0.602138000<br>H 0.602138000 0.602138000 -0.602138000<br>H -0.602138000 -0.602138000 -0.602138000<br>H 0.602138000 -0.602138000 0.602138000
NH_4^+	PBE0/def2-SVP (0 imaginary Frequencies)
	N 0.000000000 0.000000000 0.000000000<br>H -0.594478000 0.594478000 0.594478000<br>H 0.594478000 0.594478000 -0.594478000<br>H -0.594478000 -0.594478000 -0.594478000<br>H 0.594478000 -0.594478000 0.594478000

[NH_4⊂spherical cryptand]	PBE0/def2-SVP (0 imaginary Frequencies)
	N -0.832589000 2.053106000 -2.162279000<br>N -1.973252000 0.152346000 2.379829000<br>N 2.862206000 0.645642000 0.988899000<br>N 3.764520000 0.064565000 0.009372000<br>N 3.719893000 -1.445953000 -0.082852000<br>O 2.394844000 -1.881417000 -0.186881000<br>N 2.270432000 -3.260729000 -0.382945000<br>N 0.809770000 -3.658400000 -0.364222000<br>N -0.056699000 -2.851180000 -1.206234000<br>N -1.444975000 -3.277869000 -1.157687000<br>N -2.015985000 -3.437965000 0.235387000<br>O -1.731241000 -2.303957000 1.003141000<br>N -2.315069000 -2.323878000 2.273971000<br>N -1.861938000 -1.120096000 3.072555000<br>N 0.443898000 -2.717678000 -2.563960000<br>N -0.425690000 -1.891071000 -3.487596000<br>O -0.761361000 -0.679635000 -2.874060000<br>N -1.477857000 0.190511000 -3.702202000<br>N -1.895486000 1.420781000 -2.925099000<br>N 0.359282000 2.298935000 -2.956513000<br>N 1.497226000 2.964622000 -2.211957000<br>O 1.733414000 2.300255000 -1.003898000<br>N 2.837837000 2.790084000 -0.299136000<br>N 2.947226000 2.095307000 1.041469000<br>N -3.286841000 0.361243000 1.794551000<br>N -3.459704000 1.677970000 1.067440000<br>O -2.393011000 1.882646000 0.186070000<br>N -2.525714000 3.031362000 -0.601148000<br>N -1.281136000 3.231583000 -1.439758000<br>N 2.980209000 0.026836000 2.298374000<br>N 2.066790000 0.600340000 3.360970000<br>O 0.757755000 0.682333000 2.874656000<br>N -0.171683000 1.105218000 3.830590000<br>N -1.533481000 1.274678000 3.191266000<br>H 4.025428000 0.092689000 2.688582000<br>H 2.735497000 -1.041416000 2.189050000<br>H 2.113858000 -0.071238000 4.245662000<br>H 2.410950000 1.599653000 3.701552000<br>H -0.202711000 0.387176000 4.676710000<br>H 0.126020000 2.087453000 4.257215000<br>H -1.488933000 2.162964000 2.541848000<br>H -2.260077000 1.495354000 4.011252000<br>H -3.464361000 -0.447316000 1.068112000<br>H -4.092358000 0.300262000 2.566797000<br>H -4.421889000 1.634325000 0.512591000<br>H -3.538918000 2.529673000 1.775224000<br>H -3.437182000 2.959602000 -1.231264000

	H -2.651025000 3.931677000 0.038920000<br>H -0.469173000 3.540707000 -0.762866000<br>H -1.481725000 4.084548000 -2.133433000<br>H -2.442801000 -1.117275000 4.027305000<br>H -0.802243000 -1.271100000 3.331497000<br>H -2.006701000 -3.234724000 2.831253000<br>H -3.420938000 -2.364358000 2.185542000<br>H -1.623819000 -4.347465000 0.737222000<br>H -3.114387000 -3.577411000 0.136495000<br>H -2.048791000 -2.519437000 -1.680199000<br>H -1.592705000 -4.248934000 -1.690806000<br>H -2.683256000 1.117142000 -2.218071000<br>H -2.355319000 2.133182000 -3.653127000<br>H -2.400899000 -0.300499000 -4.079456000<br>H -0.871477000 0.455345000 -4.593612000<br>H -1.345990000 -2.437865000 -3.782466000<br>H 0.149785000 -1.709944000 -4.421271000<br>H 0.585768000 -3.715652000 -3.046645000<br>H 1.431343000 -2.232574000 -2.512808000<br>H 0.131662000 2.931583000 -3.849232000<br>H 0.724542000 1.327457000 -3.325103000<br>H 1.290366000 4.039033000 -2.022874000<br>H 2.397263000 2.923520000 -2.863108000<br>H 2.727278000 3.879362000 -0.107400000<br>H 3.761729000 2.665445000 -0.901993000<br>H 2.121756000 2.458648000 1.673538000<br>H 3.903141000 2.431597000 1.512838000<br>H 3.493627000 0.467404000 -0.979115000<br>H 4.825326000 0.353764000 0.209468000<br>H 4.211715000 -1.924622000 0.789779000<br>H 4.301745000 -1.746987000 -0.980725000<br>H 2.782656000 -3.818882000 0.430474000<br>H 2.764027000 -3.557728000 -1.332056000<br>H 0.756783000 -4.739709000 -0.641653000<br>H 0.452235000 -3.566125000 0.673242000<br>H -0.663639000 0.051746000 0.799732000<br>H 0.959763000 0.216573000 0.330975000<br>H -0.021085000 -0.957672000 -0.404587000<br>H -0.281214000 0.688609000 -0.726943000<br>N -0.001523000 -0.000160000 -0.000145000
[NH_4^+⊂spherical cryptand]	PBE0/def2-SVP (0 imaginary Frequencies)
	N 1.931455000 -1.828993000 -1.588715000<br>N -0.708700000 -1.344165000 2.699701000<br>N -2.588836000 0.443694000 -1.643496000<br>N -2.283494000 1.505797000 -2.588876000<br>N -2.012965000 2.856134000 -1.958308000<br>O -1.048749000 2.720741000 -0.952840000

	N	-0.605354000	3.936773000	-0.420069000
	N	0.287155000	3.673785000	0.775220000
	N	1.366280000	2.729508000	0.532337000
	N	2.213663000	2.529413000	1.697222000
	N	1.465736000	2.236768000	2.981464000
	O	0.563107000	1.187853000	2.771393000
	N	-0.060063000	0.728870000	3.937620000
	N	-1.141621000	-0.268659000	3.577757000
	N	2.124289000	3.036532000	-0.670243000
	N	3.278756000	2.096181000	-0.948307000
	O	2.827511000	0.771940000	-0.905265000
	N	3.780353000	-0.169097000	-1.312233000
	N	3.257943000	-1.567018000	-1.053082000
	N	1.797577000	-1.434023000	-2.981989000
	N	0.440099000	-1.715804000	-3.592085000
	O	-0.563192000	-1.187505000	-2.771463000
	N	-1.846121000	-1.248242000	-3.327753000
	N	-2.869622000	-0.826462000	-2.293909000
	N	0.512959000	-1.987895000	3.156105000
	N	0.986085000	-3.135427000	2.287960000
	O	1.048985000	-2.720184000	0.952759000
	N	1.631917000	-3.656366000	0.090863000
	N	1.482742000	-3.190303000	-1.342599000
	N	-3.611141000	0.822492000	-0.680874000
	N	-3.984978000	-0.263714000	0.306404000
	O	-2.827300000	-0.773066000	0.905898000
	N	-3.073428000	-1.666266000	1.955037000
	N	-1.770360000	-2.293583000	2.405355000
	H	-4.551645000	1.150118000	-1.178553000
	H	-3.227842000	1.679065000	-0.106798000
	H	-4.661144000	0.183586000	1.061997000
	H	-4.557911000	-1.078800000	-0.179106000
	H	-3.588264000	-1.148552000	2.788860000
	H	-3.749107000	-2.483326000	1.632477000
	H	-1.416891000	-2.948972000	1.595591000
	H	-2.004222000	-2.942081000	3.279628000
	H	1.307162000	-1.226753000	3.167106000
	H	0.414950000	-2.380904000	4.193181000
	H	1.983586000	-3.449110000	2.655096000
	H	0.326934000	-4.021103000	2.385181000
	H	2.694712000	-3.818424000	0.360244000
	H	1.135213000	-4.643080000	0.179618000
	H	0.414888000	-3.241732000	-1.601864000
	H	2.016262000	-3.927805000	-1.983724000
	H	-1.562232000	-0.654357000	4.533741000
	H	-1.944700000	0.279354000	3.063010000
	H	-0.538316000	1.563075000	4.488595000
	H	0.688497000	0.290124000	4.627202000

	H 0.933832000 3.132894000 3.358793000<br>H 2.212902000 1.970436000 3.755415000<br>H 2.869715000 1.671260000 1.488940000<br>H 2.868266000 3.408513000 1.892837000<br>H 3.212077000 -1.710695000 0.036643000<br>H 4.016309000 -2.278703000 -1.450543000<br>H 4.725230000 -0.050139000 -0.745438000<br>H 4.037771000 -0.019607000 -2.379826000<br>H 4.109234000 2.243634000 -0.229337000<br>H 3.685859000 2.346108000 -1.948309000<br>H 2.541022000 4.068739000 -0.648475000<br>H 1.432990000 2.979545000 -1.524065000<br>H 2.556499000 -1.927393000 -3.630215000<br>H 1.971856000 -0.349344000 -3.039503000<br>H 0.278535000 -2.801156000 -3.747858000<br>H 0.417835000 -1.251136000 -4.597960000<br>H -2.093266000 -2.280175000 -3.647495000<br>H -1.901101000 -0.619424000 -4.238761000<br>H -2.896731000 -1.601860000 -1.514032000<br>H -3.862607000 -0.826713000 -2.797470000<br>H -1.381382000 1.209040000 -3.144104000<br>H -3.099350000 1.649401000 -3.332736000<br>H -2.937317000 3.308762000 -1.547120000<br>H -1.661974000 3.538884000 -2.757586000<br>H -1.458055000 4.555551000 -0.076276000<br>H -0.084349000 4.530670000 -1.197349000<br>H 0.666963000 4.660748000 1.123412000<br>H -0.341849000 3.262125000 1.578365000<br>H -0.238661000 -0.451399000 0.905781000<br>H -0.869446000 0.149380000 -0.552989000<br>H 0.458382000 0.916673000 0.178326000<br>H 0.648304000 -0.614025000 -0.534103000<br>N -0.000353000 0.000175000 -0.000737000
[NH_4⊂[2.2.2]cryptand]	PBE0/def2-SVP (0 imaginary Frequencies)
	O 1.381106000 2.137959000 -1.356883000<br>O -1.400796000 1.893823000 -1.721520000<br>O -1.400490000 -2.439151000 -0.778019000<br>O 1.381784000 -2.244448000 -1.171931000<br>O 1.380959000 0.107094000 2.529254000<br>O -1.401049000 0.545116000 2.500454000<br>N -2.971329000 -0.000448000 -0.000620000<br>N 3.015223000 0.000101000 -0.000396000<br>N 2.739708000 0.474668000 2.460113000<br>H 3.230635000 0.216017000 3.425550000<br>H 2.831109000 1.572882000 2.340953000<br>N 3.452623000 -0.277631000 1.360625000<br>H 4.550822000 -0.090483000 1.466529000

	H	3.303654000	-1.348632000	1.563930000
	N	2.739606000	1.894020000	-1.641296000
	H	3.230770000	2.859234000	-1.900405000
	H	2.829845000	1.241601000	-2.532808000
	N	3.452944000	1.317563000	-0.440439000
	H	4.551051000	1.315320000	-0.655991000
	H	3.304708000	2.029235000	0.385451000
	N	2.740482000	-2.368111000	-0.819001000
	H	3.231583000	-3.075047000	-1.525443000
	H	2.831721000	-2.813638000	0.191853000
	N	3.453099000	-1.039744000	-0.921132000
	H	4.551271000	-1.224861000	-0.811476000
	H	3.304541000	-0.680631000	-1.950460000
	N	0.697858000	0.700523000	3.608172000
	H	0.695938000	1.804023000	3.502128000
	H	1.218317000	0.446953000	4.559949000
	N	-0.713723000	0.193303000	3.670371000
	H	-0.708794000	-0.908116000	3.804530000
	H	-1.216447000	0.640105000	4.560003000
	N	0.697770000	2.776667000	-2.409468000
	H	0.695873000	2.134167000	-3.312869000
	H	1.218017000	3.728123000	-2.664759000
	N	-0.713810000	3.083355000	-2.000751000
	H	-0.709021000	3.749432000	-1.113356000
	H	-1.216742000	3.630957000	-2.832019000
	N	0.698808000	-3.475611000	-1.196999000
	H	0.697022000	-3.935358000	-0.188226000
	H	1.219328000	-4.173201000	-1.892342000
	N	-0.712750000	-3.276207000	-1.667540000
	H	-0.707824000	-2.841648000	-2.688458000
	H	-1.215142000	-4.270219000	-1.725465000
	N	-2.750847000	2.089810000	-1.400042000
	H	-3.269622000	2.591533000	-2.249136000
	H	-2.847937000	2.769873000	-0.529266000
	N	-3.432591000	0.763595000	-1.160351000
	H	-4.533944000	0.935083000	-1.088592000
	H	-3.268793000	0.156731000	-2.062794000
	N	-2.750594000	0.166925000	2.509287000
	H	-3.270105000	0.650386000	3.368442000
	H	-2.846144000	-0.927424000	2.662374000
	N	-3.432722000	0.621876000	1.240881000
	H	-4.533983000	0.473504000	1.353617000
	H	-3.269402000	1.706919000	1.166303000
	N	-2.749986000	-2.258041000	-1.110323000
	H	-3.269246000	-3.243955000	-1.121008000
	H	-2.845422000	-1.843856000	-2.134784000
	N	-3.432408000	-1.386933000	-0.082394000
	H	-4.533648000	-1.410668000	-0.267353000

	H -3.269043000 -1.864841000 0.894572000<br>H 0.388914000 0.808347000 -0.526937000<br>H 0.389063000 -0.860601000 -0.436379000<br>H 0.388378000 0.052460000 0.963541000<br>H -1.020583000 -0.000250000 -0.000570000<br>N 0.021301000 -0.000046000 -0.000120000
[NH_4^+⊂[2.2.2]cryptand]	PBE0/def2-SVP (0 imaginary Frequencies)
	O -1.396151000 2.466690000 0.581231000<br>O 1.418494000 2.413830000 0.896626000<br>O 1.418980000 -1.983523000 1.641509000<br>O -1.395744000 -1.737017000 1.845404000<br>O -1.395689000 -0.729702000 -2.426322000<br>O 1.418765000 -0.430247000 -2.537990000<br>N 2.993216000 0.000387000 -0.000041000<br>N -3.044062000 -0.000097000 0.000094000<br>N -2.707651000 -0.220920000 -2.479524000<br>H -3.232885000 -0.637461000 -3.360134000<br>H -2.686602000 0.878205000 -2.615019000<br>N -3.482211000 -0.621670000 -1.241869000<br>H -4.560159000 -0.425887000 -1.434083000<br>H -3.381855000 -1.712924000 -1.145741000<br>N -2.707971000 2.257891000 1.048537000<br>H -3.233545000 3.228591000 1.128227000<br>H -2.686590000 1.825556000 2.068104000<br>N -3.482296000 1.386207000 0.082600000<br>H -4.560299000 1.454684000 0.348070000<br>H -3.381800000 1.848489000 -0.910594000<br>N -2.707487000 -2.037308000 1.430665000<br>H -3.232989000 -2.592331000 2.231067000<br>H -2.685917000 -2.703495000 0.546032000<br>N -3.482014000 -0.764957000 1.159381000<br>H -4.560037000 -1.029076000 1.086188000<br>H -3.381274000 -0.136201000 2.056421000<br>N -0.680596000 -0.567301000 -3.627934000<br>H -0.591621000 0.505592000 -3.886883000<br>H -1.208804000 -1.071568000 -4.459352000<br>N 0.691771000 -1.166840000 -3.481894000<br>H 0.601989000 -2.227594000 -3.172850000<br>H 1.193675000 -1.151245000 -4.469648000<br>N -0.681133000 3.426030000 1.322894000<br>H -0.592223000 3.113602000 2.381465000<br>H -1.209412000 4.398153000 1.302109000<br>N 0.691342000 3.599538000 0.730835000<br>H 0.601627000 3.862482000 -0.342286000<br>H 1.193130000 4.447122000 1.238386000<br>N -0.680509000 -2.859231000 2.304492000<br>H -0.591528000 -3.619172000 1.504076000

	H -1.208693000 -3.328002000 3.156438000<br>N 0.691943000 -2.433478000 2.751039000<br>H 0.602319000 -1.636066000 3.515803000<br>H 1.193776000 -3.297128000 3.230701000<br>N 2.722489000 2.474684000 0.387697000<br>H 3.279726000 3.312662000 0.851820000<br>H 2.705852000 2.676319000 -0.702203000<br>N 3.458585000 1.192475000 0.710213000<br>H 4.542452000 1.354848000 0.531245000<br>H 3.342619000 1.024808000 1.790799000<br>N 2.722825000 -0.901247000 -2.336395000<br>H 3.279974000 -0.918310000 -3.294219000<br>H 2.706196000 -1.945919000 -1.965990000<br>N 3.458941000 0.019273000 -1.387407000<br>H 4.542769000 -0.217194000 -1.438262000<br>H 3.343299000 1.038855000 -1.782783000<br>N 2.722832000 -1.572839000 1.948831000<br>H 3.280314000 -2.393701000 2.442311000<br>H 2.705934000 -0.729940000 2.668615000<br>N 3.458771000 -1.210713000 0.677159000<br>H 4.542646000 -1.136749000 0.907197000<br>H 3.342900000 -2.062732000 -0.008343000<br>H -0.385543000 0.939332000 0.223977000<br>H -0.385344000 -0.662978000 0.701860000<br>H -0.385848000 -0.275568000 -0.924691000<br>H 1.025656000 0.000293000 0.000027000<br>N -0.017567000 0.000259000 0.000312000
[NH_4⊂[bpy.bpy.bpy]cryptand]	PBE0/def2-SVP (0 imaginary Frequencies)
	N -2.880016000 0.029400000 -0.040920000<br>N 2.859012000 -0.031539000 0.049270000<br>N 3.262870000 -1.377044000 -0.321278000<br>H 4.368621000 -1.491287000 -0.277680000<br>H 2.972592000 -1.527969000 -1.374434000<br>N 3.246998000 0.309007000 1.407612000<br>H 4.353578000 0.362023000 1.509529000<br>H 2.910488000 -0.512330000 2.062073000<br>N 3.297515000 0.962204000 -0.916387000<br>H 4.406860000 0.983410000 -0.996481000<br>H 2.990906000 1.949780000 -0.533275000<br>N -3.268796000 1.420726000 0.043631000<br>H -4.375952000 1.537081000 0.092212000<br>H -2.947459000 1.911199000 -0.890970000<br>N -3.304283000 -0.741200000 1.108392000<br>H -4.413640000 -0.836361000 1.154415000<br>H -3.006428000 -0.181850000 2.011739000<br>N -3.261722000 -0.583424000 -1.295812000<br>H -4.366852000 -0.577503000 -1.437616000<br>H -2.961949000 -1.644245000 -1.249931000

	N	2.629941000	-2.494884000	0.485999000
	N	1.306358000	-2.500447000	0.601109000
	N	3.432187000	-3.510991000	1.019828000
	N	0.683927000	-3.506999000	1.239086000
	N	2.801801000	-4.569037000	1.682508000
	N	1.421336000	-4.574697000	1.793631000
	H	4.518883000	-3.473470000	0.919710000
	H	3.391840000	-5.378197000	2.120307000
	H	0.921612000	-5.381761000	2.331155000
	N	-0.789705000	-3.447681000	1.339167000
	N	-1.385030000	-2.254533000	1.144665000
	N	-1.563615000	-4.590402000	1.628158000
	N	-2.707818000	-2.131733000	1.229514000
	N	-2.941289000	-4.474624000	1.702059000
	N	-3.537566000	-3.224490000	1.499704000
	H	-1.090411000	-5.563909000	1.762500000
	H	-3.556576000	-5.354515000	1.906009000
	H	-4.620930000	-3.099089000	1.549381000
	N	2.712624000	0.818376000	-2.308662000
	N	1.391630000	0.723232000	-2.412350000
	N	3.553231000	0.860159000	-3.427080000
	N	0.806770000	0.674527000	-3.623890000
	N	2.962863000	0.815459000	-4.695685000
	N	1.585599000	0.725884000	-4.800945000
	H	4.636741000	0.925826000	-3.308747000
	H	3.582533000	0.837923000	-5.595746000
	H	1.117995000	0.664134000	-5.784654000
	N	-0.663218000	0.561082000	-3.670787000
	N	-1.298261000	0.135253000	-2.558611000
	N	-1.400439000	0.880703000	-4.831104000
	N	-2.622982000	0.004454000	-2.540490000
	N	-2.778768000	0.762062000	-4.814035000
	N	-3.416199000	0.315798000	-3.648600000
	H	-0.896350000	1.248386000	-5.725837000
	H	-3.364085000	1.024634000	-5.698843000
	H	-4.501830000	0.211167000	-3.600888000
	N	2.648877000	1.593183000	1.950556000
	N	1.329552000	1.734293000	1.881805000
	N	3.472548000	2.548350000	2.558689000
	N	0.732693000	2.814075000	2.414159000
	N	2.868660000	3.677318000	3.121214000
	N	1.491951000	3.815708000	3.054786000
	H	4.555509000	2.411888000	2.589184000
	H	3.476499000	4.450714000	3.597585000
	H	1.012794000	4.705013000	3.466730000
	N	-0.738210000	2.910599000	2.296366000
	N	-1.341894000	2.164415000	1.350970000

	N -1.498284000 3.750794000 3.135012000<br>N -2.662674000 2.208360000 1.190656000<br>N -2.873961000 3.793550000 2.982929000<br>N -3.479927000 3.009594000 1.994630000<br>H -1.016914000 4.338967000 3.917430000<br>H -3.480118000 4.425325000 3.636996000<br>H -4.561968000 3.018293000 1.849612000<br>H -0.387240000 0.005311000 -0.980143000<br>H -0.396274000 0.844779000 0.482007000<br>H -0.413677000 -0.841905000 0.478990000<br>H 0.982365000 -0.011728000 0.011162000<br>N -0.046888000 -0.001261000 -0.000892000
[NH_4^+⊂[bpy.bpy.bpy]cryptand]	PBE0/def2-SVP (0 imaginary Frequencies)
	N 0.000000000 0.000000000 2.910956000<br>N 0.000000000 0.000000000 -2.890426000<br>N -1.370606000 0.256805000 -3.313407000<br>H -1.466772000 0.204355000 -4.418721000<br>H -1.619517000 1.290869000 -3.023633000<br>N 0.462903000 -1.315382000 -3.313407000<br>H 0.556409000 -1.372439000 -4.418721000<br>H -0.308167000 -2.047977000 -3.023633000<br>N 0.907703000 1.058577000 -3.313407000<br>H 0.910363000 1.168084000 -4.418721000<br>H 1.927684000 0.757108000 -3.023633000<br>N 1.386807000 -0.112883000 3.322598000<br>H 1.482909000 -0.199238000 4.427511000<br>H 1.896833000 0.823639000 3.040564000<br>N -0.791163000 -1.144569000 3.322598000<br>H -0.914000000 -1.184618000 4.427511000<br>H -0.235124000 -2.054525000 3.040564000<br>N -0.595644000 1.257451000 3.322598000<br>H -0.568909000 1.383856000 4.427511000<br>H -1.661709000 1.230886000 3.040564000<br>N -2.410632000 -0.649430000 -2.687938000<br>N -2.473896000 -0.682943000 -1.357893000<br>N -3.301605000 -1.374384000 -3.487463000<br>N -3.406646000 -1.418573000 -0.752699000<br>N -4.281312000 -2.144022000 -2.866544000<br>N -4.342064000 -2.170230000 -1.478115000<br>H -3.226186000 -1.336217000 -4.576391000<br>H -4.987031000 -2.728467000 -3.461443000<br>H -5.085324000 -2.785534000 -0.968707000<br>N -3.410803000 -1.417026000 0.741894000<br>N -2.237108000 -1.244814000 1.355605000<br>N -4.595915000 -1.595840000 1.468021000<br>N -2.163276000 -1.248968000 2.687630000<br>N -4.534898000 -1.588374000 2.856440000<br>N -3.303066000 -1.415006000 3.481004000

	H -5.552372000 -1.711455000 0.956173000<br>H -5.444291000 -1.709922000 3.449572000<br>H -3.222919000 -1.407154000 4.570065000<br>N 0.642893000 2.412383000 -2.687938000<br>N 0.645502000 2.483929000 -1.357893000<br>N 0.460551000 3.546466000 -3.487463000<br>N 0.474803000 3.659528000 -0.752699000<br>N 0.283878000 4.779736000 -2.866544000<br>N 0.291558000 4.845453000 -1.478115000<br>H 0.455895000 3.462067000 -4.576391000<br>H 0.130594000 5.683129000 -3.461443000<br>H 0.130318000 5.796787000 -0.968707000<br>N 0.478221000 3.662355000 0.741894000<br>N 0.040514000 2.559800000 1.355605000<br>N 0.915920000 4.778100000 1.468021000<br>N 0.000000000 2.497935000 2.687630000<br>N 0.891877000 4.721524000 2.856440000<br>N 0.426102000 3.568043000 3.481004000<br>H 1.294023000 5.664223000 0.956173000<br>H 1.241310000 5.569855000 3.449572000<br>H 0.392829000 3.494706000 4.570065000<br>N 1.767739000 -1.762953000 -2.687938000<br>N 1.828394000 -1.800985000 -1.357893000<br>N 2.841054000 -2.172082000 -3.487463000<br>N 2.931843000 -2.240955000 -0.752699000<br>N 3.997434000 -2.635714000 -2.866544000<br>N 4.050506000 -2.675223000 -1.478115000<br>H 2.770291000 -2.125850000 -4.576391000<br>H 4.856437000 -2.954663000 -3.461443000<br>H 4.955006000 -3.011252000 -0.968707000<br>N 2.932582000 -2.245329000 0.741894000<br>N 2.196595000 -1.314986000 1.355605000<br>N 3.679996000 -3.182260000 1.468021000<br>N 2.163276000 -1.248968000 2.687630000<br>N 3.643021000 -3.133150000 2.856440000<br>N 2.876965000 -2.153036000 3.481004000<br>H 4.258350000 -3.952768000 0.956173000<br>H 4.202981000 -3.859933000 3.449572000<br>H 2.830090000 -2.087553000 4.570065000<br>H -0.060547000 0.969986000 0.392014000<br>H 0.870306000 -0.432558000 0.392014000<br>H -0.809759000 -0.537428000 0.392014000<br>H 0.000000000 0.000000000 -0.996228000<br>N 0.000000000 0.000000000 0.036287000
H_3O	PBE0/def2-SVP (0 imaginary Frequencies)
	O -0.000010000 0.000000000 0.112273000<br>H 0.959241000 0.000000000 -0.240750000

	H -0.479618000 -0.830794000 -0.240718000<br>H -0.479618000 0.830794000 -0.240718000
H_3O^+	PBE0/def2-SVP (0 imaginary Frequencies)
	O -0.000027000 0.000000000 0.052278000<br>H 0.942890000 0.000000000 -0.220791000<br>H -0.471432000 -0.816648000 -0.220699000<br>H -0.471432000 0.816648000 -0.220699000
[H_3O⊂spherical cryptand]	PBE0/def2-SVP (0 imaginary Frequencies)
	N -1.012363000 -1.456337000 -2.373766000<br>N -1.037591000 2.777305000 -0.085509000<br>N 3.289816000 0.003433000 0.027006000<br>N 3.593095000 -1.274139000 0.612699000<br>N 3.143737000 -1.439555000 2.051505000<br>O 1.788006000 -1.133484000 2.169098000<br>N 1.253875000 -1.386684000 3.431620000<br>N -0.170555000 -0.872867000 3.507275000<br>N -1.055467000 -1.323284000 2.434587000<br>N -2.435362000 -0.868580000 2.621974000<br>N -2.583010000 0.588803000 2.989282000<br>O -1.675288000 1.348832000 2.239136000<br>N -1.929113000 2.719412000 2.236454000<br>N -0.992156000 3.368935000 1.247329000<br>N -0.996102000 -2.773123000 2.282419000<br>N -1.915746000 -3.313372000 1.214682000<br>O -1.650891000 -2.631186000 0.028283000<br>N -2.545189000 -2.906294000 -1.015139000<br>N -2.392661000 -1.857736000 -2.091204000<br>N -0.116780000 -2.603387000 -2.511532000<br>N 1.309718000 -2.269225000 -2.901725000<br>O 1.825321000 -1.297853000 -2.045085000<br>N 3.180751000 -1.029836000 -2.236029000<br>N 3.608550000 0.138004000 -1.368518000<br>N -2.411355000 2.719013000 -0.590474000<br>N -2.542400000 2.309062000 -2.038007000<br>O -1.635097000 1.276082000 -2.309331000<br>N -1.876865000 0.588495000 -3.497432000<br>N -0.945410000 -0.597812000 -3.551645000<br>N 3.587323000 1.147800000 0.845049000<br>N 3.154213000 2.479021000 0.262081000<br>O 1.803004000 2.436047000 -0.081240000<br>N 1.281915000 3.659790000 -0.498624000<br>N -0.137534000 3.477884000 -0.999454000<br>H 4.676276000 1.247509000 1.083911000<br>H 3.054704000 1.027633000 1.800481000<br>H 3.346538000 3.260142000 1.027340000<br>H 3.769216000 2.756207000 -0.619877000<br>H 1.333933000 4.403996000 0.323404000<br>H 1.877226000 4.084510000 -1.334281000

	H -0.104573000 2.899640000 -1.932435000<br>H -0.528145000 4.490458000 -1.243835000<br>H -2.965551000 1.945669000 -0.004625000<br>H -2.924374000 3.701460000 -0.463944000<br>H -3.583725000 1.930092000 -2.174009000<br>H -2.382982000 3.154754000 -2.738549000<br>H -2.941024000 0.278909000 -3.544515000<br>H -1.686291000 1.230767000 -4.383262000<br>H 0.084170000 -0.221762000 -3.640357000<br>H -1.178134000 -1.176760000 -4.475094000<br>H -1.235274000 4.455827000 1.204918000<br>H 0.034023000 3.268251000 1.629708000<br>H -1.754042000 3.165906000 3.238187000<br>H -2.992156000 2.905620000 1.979985000<br>H -2.435428000 0.774215000 4.073372000<br>H -3.624673000 0.886609000 2.719590000<br>H -2.978756000 -0.994439000 1.653774000<br>H -2.951184000 -1.473766000 3.404222000<br>H -2.947848000 -0.961053000 -1.722184000<br>H -2.893812000 -2.236048000 -3.013099000<br>H -3.591620000 -2.828314000 -0.633752000<br>H -2.386768000 -3.936577000 -1.395766000<br>H -2.983270000 -3.190911000 1.490219000<br>H -1.731722000 -4.403177000 1.105271000<br>H -1.246432000 -3.280978000 3.242426000<br>H 0.035504000 -3.045577000 2.016243000<br>H -0.502279000 -3.319230000 -3.270762000<br>H -0.099454000 -3.121853000 -1.543638000<br>H 1.377179000 -1.931072000 -3.956894000<br>H 1.899065000 -3.208554000 -2.842992000<br>H 3.388824000 -0.758641000 -3.292412000<br>H 3.787832000 -1.935749000 -2.026936000<br>H 3.086757000 1.028617000 -1.749935000<br>H 4.701279000 0.288440000 -1.558460000<br>H 3.075529000 -2.046799000 0.024535000<br>H 4.684888000 -1.519827000 0.593008000<br>H 3.744254000 -0.808724000 2.740240000<br>H 3.342381000 -2.491104000 2.347441000<br>H 1.835893000 -0.869870000 4.223629000<br>H 1.311536000 -2.470015000 3.666977000<br>H -0.570224000 -1.168462000 4.502497000<br>H -0.145812000 0.224500000 3.470924000<br>H -0.630881000 0.973856000 -0.002512000<br>H -0.638265000 -0.493350000 0.839045000<br>H -0.621698000 -0.488762000 -0.853070000<br>O -0.357430000 -0.002398000 -0.002849000
[H_3O^+⊂spherical cryptand]	PBE0/def2-SVP (0 imaginary Frequencies)

	N	-1.035817000	0.719248000	-2.670137000
	N	-1.042529000	1.949451000	1.958777000
	N	3.317793000	0.007006000	0.002353000
	N	3.644067000	-1.278596000	-0.558329000
	N	3.191502000	-2.463153000	0.270537000
	O	1.826218000	-2.348142000	0.565535000
	N	1.310837000	-3.429765000	1.275194000
	N	-0.093282000	-3.116107000	1.747424000
	N	-1.030528000	-2.674958000	0.709214000
	N	-2.401859000	-2.631834000	1.217800000
	N	-2.543587000	-1.873197000	2.520389000
	O	-1.886026000	-0.648246000	2.380430000
	N	-1.936550000	0.197926000	3.492008000
	N	-0.941876000	1.317997000	3.273861000
	N	-0.928596000	-3.498185000	-0.495023000
	N	-1.925818000	-3.131089000	-1.573120000
	O	-1.880436000	-1.745234000	-1.750454000
	N	-2.540815000	-1.256533000	-2.880717000
	N	-2.405362000	0.251389000	-2.886335000
	N	-0.096053000	0.044953000	-3.571953000
	N	1.305553000	0.616965000	-3.608271000
	O	1.822212000	0.691463000	-2.317263000
	N	3.186401000	1.009394000	-2.270689000
	N	3.639636000	1.136595000	-0.830798000
	N	-2.414166000	2.365208000	1.663984000
	N	-2.554396000	3.112882000	0.354897000
	O	-1.892041000	2.380600000	-0.633815000
	N	-1.940625000	2.919607000	-1.922709000
	N	-0.940519000	2.174274000	-2.780495000
	N	3.638343000	0.164788000	1.397348000
	N	3.180608000	1.473807000	2.007122000
	O	1.815634000	1.668449000	1.755531000
	N	1.295680000	2.822851000	2.335315000
	N	-0.107537000	3.071265000	1.823262000
	H	4.731646000	0.080003000	1.611473000
	H	3.138270000	-0.642217000	1.953642000
	H	3.381288000	1.432818000	3.096902000
	H	3.765097000	2.331446000	1.617395000
	H	1.318426000	2.755397000	3.442423000
	H	1.901477000	3.714103000	2.072204000
	H	-0.042356000	3.318835000	0.754001000
	H	-0.490560000	3.968235000	2.354803000
	H	-3.033197000	1.458029000	1.609203000
	H	-2.827412000	3.007519000	2.470563000
	H	-3.632478000	3.224521000	0.125603000
	H	-2.137546000	4.136787000	0.421036000
	H	-2.968338000	2.859923000	-2.332175000
	H	-1.664377000	3.992223000	-1.920088000

	H 0.070345000 2.458240000 -2.455333000<br>H -1.069876000 2.513770000 -3.829982000<br>H -1.072883000 2.056390000 4.093031000<br>H 0.071025000 0.898896000 3.354961000<br>H -1.656368000 -0.339629000 4.419015000<br>H -2.965687000 0.577869000 3.646746000<br>H -2.123901000 -2.441194000 3.373512000<br>H -3.622007000 -1.734543000 2.732753000<br>H -3.022620000 -2.131895000 0.460259000<br>H -2.812133000 -3.652773000 1.370285000<br>H -3.027417000 0.654823000 -2.074268000<br>H -2.817715000 0.628170000 -3.846482000<br>H -3.618158000 -1.514265000 -2.866069000<br>H -2.119912000 -1.709447000 -3.799563000<br>H -2.953565000 -3.458708000 -1.320431000<br>H -1.644647000 -3.664328000 -2.502322000<br>H -1.055703000 -4.577144000 -0.264540000<br>H 0.083312000 -3.355665000 -0.899966000<br>H -0.476216000 0.056878000 -4.615566000<br>H -0.028317000 -1.005033000 -3.252931000<br>H 1.326552000 1.610404000 -4.101640000<br>H 1.914872000 -0.053611000 -4.248280000<br>H 3.386670000 1.974138000 -2.779337000<br>H 3.773862000 0.244243000 -2.817054000<br>H 3.137268000 2.021366000 -0.411790000<br>H 4.732766000 1.365751000 -0.861147000<br>H 3.144516000 -1.359116000 -1.535480000<br>H 4.738016000 -1.416890000 -0.738631000<br>H 3.776675000 -2.552360000 1.207939000<br>H 3.395712000 -3.385517000 -0.310084000<br>H 1.917986000 -3.645661000 2.178136000<br>H 1.336851000 -4.354428000 0.662751000<br>H -0.472739000 -4.026745000 2.257874000<br>H -0.030289000 -2.314484000 2.497203000<br>H -0.662010000 0.691135000 0.700896000<br>H -0.656801000 -0.955328000 0.248210000<br>H -0.658613000 0.260001000 -0.951454000<br>O -0.414405000 -0.000743000 -0.000449000
[H_3O⊂[2.2.2]cryptand]	PBE0/def2-SVP (0 imaginary Frequencies)
	O -1.246393000 -1.329531000 1.948162000<br>O 1.709164000 -1.309277000 1.928974000<br>O 1.397865000 -1.323797000 -2.209243000<br>O -1.209727000 -0.623460000 -2.223014000<br>O -1.515981000 2.358006000 0.239571000<br>O 1.367657000 2.608818000 0.081495000<br>N 3.232329000 0.178974000 -0.140759000<br>N -3.145747000 -0.050275000 0.045765000

	N	-2.759348000	2.240118000	0.903185000
	H	-3.240210000	3.235014000	0.962123000
	H	-2.604955000	1.884507000	1.939997000
	N	-3.655532000	1.300628000	0.119365000
	H	-4.678239000	1.346661000	0.555623000
	H	-3.739551000	1.713238000	-0.897893000
	N	-2.487259000	-1.922789000	1.564967000
	H	-2.876021000	-2.499337000	2.431915000
	H	-2.274314000	-2.620714000	0.703431000
	N	-3.482719000	-0.844667000	1.217903000
	H	-4.484562000	-1.321601000	1.107080000
	H	-3.564936000	-0.162825000	2.081262000
	N	-2.565257000	-0.244475000	-2.361894000
	H	-2.962011000	-0.646966000	-3.309982000
	H	-2.577396000	0.855396000	-2.449902000
	N	-3.432757000	-0.722274000	-1.209676000
	H	-4.507326000	-0.629880000	-1.493552000
	H	-3.214020000	-1.805748000	-1.085984000
	N	-0.771216000	3.509793000	0.564711000
	H	-0.467037000	3.496427000	1.628653000
	H	-1.391968000	4.410244000	0.397613000
	N	0.450750000	3.578823000	-0.313420000
	H	0.135078000	3.434520000	-1.366940000
	H	0.886189000	4.598187000	-0.240700000
	N	-0.369878000	-2.283262000	2.538609000
	H	-0.132348000	-3.063728000	1.780228000
	H	-0.894686000	-2.740116000	3.403526000
	N	0.882562000	-1.600499000	3.014780000
	H	0.607894000	-0.676805000	3.567075000
	H	1.400615000	-2.267457000	3.737735000
	N	-0.856496000	-1.861101000	-2.834800000
	H	-0.831345000	-1.714494000	-3.929592000
	H	-1.616136000	-2.637023000	-2.552534000
	N	0.446515000	-2.351030000	-2.273766000
	H	0.216000000	-2.751483000	-1.246394000
	H	0.801699000	-3.197154000	-2.904980000
	N	2.786687000	-0.471920000	2.219353000
	H	3.330601000	-0.828518000	3.120237000
	H	2.436730000	0.559224000	2.435381000
	N	3.743976000	-0.474929000	1.043484000
	H	4.713193000	-0.038621000	1.371822000
	H	3.951628000	-1.530809000	0.810816000
	N	2.428903000	2.439743000	-0.823945000
	H	2.859556000	3.428585000	-1.085706000
	H	2.062300000	1.979125000	-1.762433000
	N	3.520260000	1.590698000	-0.201827000
	H	4.470519000	1.798518000	-0.746344000
	H	3.667342000	1.972767000	0.820859000

	N 2.589974000 -1.738019000 -1.599866000<br>H 3.111339000 -2.469892000 -2.254587000<br>H 2.372446000 -2.247427000 -0.640481000<br>N 3.493415000 -0.539263000 -1.371326000<br>H 4.556748000 -0.867278000 -1.431458000<br>H 3.336365000 0.139079000 -2.225027000<br>H -0.561797000 -0.597267000 0.731871000<br>H -0.523289000 -0.304055000 -0.887089000<br>H -0.640808000 0.911042000 0.146592000<br>O -0.191940000 0.034531000 0.017514000
[H_3O^+⊂[2.2.2]cryptand]	PBE0/def2-SVP (0 imaginary Frequencies)
	O 1.391238000 1.104132000 2.085070000<br>O -1.477543000 1.207260000 2.173066000<br>O -1.478791000 1.278624000 -2.131938000<br>O 1.390272000 1.253940000 -1.998579000<br>O 1.390737000 -2.357965000 -0.086717000<br>O -1.478363000 -2.486605000 -0.040410000<br>N -3.207149000 -0.000132000 0.000271000<br>N 3.181581000 -0.000046000 -0.000425000<br>N 2.683637000 -2.387918000 0.490255000<br>H 3.117660000 -3.394393000 0.355678000<br>H 2.616664000 -2.207981000 1.580026000<br>N 3.582192000 -1.383198000 -0.196688000<br>H 4.627480000 -1.564136000 0.133385000<br>H 3.557485000 -1.610824000 -1.273055000<br>N 2.683942000 1.618991000 1.821985000<br>H 3.118169000 2.006083000 2.760644000<br>H 2.616510000 2.472547000 1.121014000<br>N 3.582518000 0.521672000 1.295390000<br>H 4.627803000 0.898003000 1.286726000<br>H 3.557946000 -0.296573000 2.030854000<br>N 2.682962000 0.769040000 -2.313548000<br>H 3.116798000 1.388757000 -3.118045000<br>H 2.615653000 -0.264660000 -2.702652000<br>N 3.582016000 0.861404000 -1.100287000<br>H 4.627174000 0.665836000 -1.422339000<br>H 3.557603000 1.907402000 -0.759247000<br>N 0.630345000 -3.531684000 0.159369000<br>H 0.429501000 -3.637092000 1.241197000<br>H 1.208497000 -4.411777000 -0.173251000<br>N -0.667447000 -3.472224000 -0.596472000<br>H -0.459452000 -3.272032000 -1.668090000<br>H -1.146642000 -4.471550000 -0.542028000<br>N 0.630741000 1.904233000 2.978279000<br>H 0.429671000 2.893643000 2.528344000<br>H 1.208889000 2.056649000 3.906709000<br>N -0.666880000 1.219662000 3.304859000

	H -0.458580000 0.191872000 3.668126000<br>H -1.146494000 1.766906000 4.142556000<br>N 0.629611000 1.628359000 -3.137677000<br>H 0.428497000 0.744519000 -3.770279000<br>H 1.207661000 2.356708000 -3.733358000<br>N -0.668009000 2.253096000 -2.707542000<br>H -0.459711000 3.080857000 -1.998224000<br>H -1.147539000 2.705874000 -3.599891000<br>N -2.703616000 0.552011000 2.376083000<br>H -3.211199000 0.969407000 3.268951000<br>H -2.533627000 -0.524720000 2.575581000<br>N -3.604331000 0.746610000 1.175865000<br>H -4.649311000 0.514889000 1.481302000<br>H -3.589960000 1.821901000 0.941230000<br>N -2.704049000 -2.333629000 -0.709831000<br>H -3.212047000 -3.315255000 -0.795893000<br>H -2.533317000 -1.967127000 -1.741613000<br>N -3.604549000 -1.391562000 0.059210000<br>H -4.649621000 -1.539969000 -0.293982000<br>H -3.590052000 -1.726237000 1.107682000<br>N -2.705021000 1.781564000 -1.665888000<br>H -3.212973000 2.345785000 -2.473797000<br>H -2.535289000 2.492893000 -0.833268000<br>N -3.605159000 0.644495000 -1.234005000<br>H -4.650210000 1.024599000 -1.185454000<br>H -3.591045000 -0.096269000 -2.047992000<br>H 0.617198000 0.449183000 0.823163000<br>H 0.616741000 0.487638000 -0.800459000<br>H 0.617126000 -0.937798000 -0.021995000<br>O 0.247688000 -0.000368000 0.000342000
[H_3O⊂[bpy.bpy.bpy]cryptand]	PBE0/def2-SVP (0 imaginary Frequencies)
	N -2.870429000 -0.190018000 0.120643000<br>N 2.857550000 0.205301000 -0.124227000<br>N 3.176146000 1.231402000 0.831336000<br>H 4.273538000 1.429715000 0.892287000<br>H 2.869070000 0.869025000 1.826762000<br>N 3.120545000 0.568096000 -1.490441000<br>H 4.211012000 0.690347000 -1.699255000<br>H 2.655666000 1.550965000 -1.675134000<br>N 3.338494000 -1.100798000 0.239724000<br>H 4.453833000 -1.157329000 0.244556000<br>H 2.994794000 -1.812468000 -0.529607000<br>N -3.179435000 -1.506591000 -0.375557000<br>H -4.276392000 -1.694608000 -0.420351000<br>H -2.768136000 -2.240861000 0.338062000<br>N -3.362748000 0.873237000 -0.717355000<br>H -4.475190000 0.932497000 -0.713607000<br>H -3.071789000 0.648079000 -1.758016000

	N	-3.175984000	-0.009745000	1.518459000
	H	-4.267397000	-0.091403000	1.723687000
	H	-2.884198000	1.017665000	1.796181000
	N	2.475454000	2.553141000	0.600108000
	N	1.155770000	2.540940000	0.444131000
	N	3.215258000	3.742625000	0.592668000
	N	0.487966000	3.687393000	0.259397000
	N	2.533149000	4.947506000	0.414265000
	N	1.158964000	4.928174000	0.241142000
	H	4.300048000	3.719416000	0.715204000
	H	3.076562000	5.895618000	0.394239000
	H	0.622542000	5.861759000	0.070041000
	N	-0.971874000	3.588573000	0.061158000
	N	-1.503838000	2.364143000	-0.161581000
	N	-1.794845000	4.731769000	0.092645000
	N	-2.816480000	2.243010000	-0.379873000
	N	-3.155618000	4.604944000	-0.116653000
	N	-3.686458000	3.334329000	-0.361667000
	H	-1.368639000	5.712718000	0.301602000
	H	-3.804815000	5.483090000	-0.081379000
	H	-4.754844000	3.187468000	-0.529804000
	N	2.834340000	-1.609590000	1.573635000
	N	1.526493000	-1.558504000	1.797982000
	N	3.734269000	-2.146175000	2.502258000
	N	1.019990000	-2.020397000	2.952461000
	N	3.222665000	-2.647547000	3.703387000
	N	1.860661000	-2.584925000	3.938945000
	H	4.805478000	-2.163202000	2.291267000
	H	3.891567000	-3.069138000	4.458155000
	H	1.459736000	-2.944879000	4.886884000
	N	-0.431305000	-1.893156000	3.144171000
	N	-1.131106000	-1.117665000	2.273037000
	N	-1.093228000	-2.543787000	4.207112000
	N	-2.447588000	-0.954648000	2.447311000
	N	-2.454712000	-2.388352000	4.374039000
	N	-3.157408000	-1.570961000	3.475836000
	H	-0.535701000	-3.187867000	4.887021000
	H	-2.975164000	-2.900585000	5.186714000
	H	-4.233875000	-1.417141000	3.568795000
	N	2.552225000	-0.395280000	-2.510281000
	N	1.261149000	-0.698397000	-2.422638000
	N	3.371417000	-0.913453000	-3.521374000
	N	0.704038000	-1.533265000	-3.307969000
	N	2.803200000	-1.777270000	-4.458931000
	N	1.459679000	-2.099661000	-4.355294000
	H	4.430853000	-0.652114000	-3.562056000
	H	3.412977000	-2.210794000	-5.255692000

	H	1.014351000	-2.801216000	-5.061274000
	N	-0.728410000	-1.846477000	-3.124205000
	N	-1.304638000	-1.519807000	-1.945075000
	N	-1.474895000	-2.475786000	-4.138375000
	N	-2.590704000	-1.814584000	-1.733943000
	N	-2.809546000	-2.771002000	-3.925545000
	N	-3.386858000	-2.437256000	-2.696755000
	H	-1.013512000	-2.709791000	-5.097706000
	H	-3.403088000	-3.246867000	-4.709797000
	H	-4.436672000	-2.649213000	-2.486077000
	H	-0.401351000	-0.444316000	0.908320000
	H	-0.441421000	-0.571068000	-0.736406000
	H	-0.517952000	0.907872000	-0.036870000
	O	-0.110092000	-0.010537000	0.031539000
[H_3O^+⊂[bpy.bpy.bpy]cryptand]	PBE0/def2-SVP (0 imaginary Frequencies)			
	N	-2.912623000	0.000150000	0.000746000
	N	2.906129000	-0.000175000	-0.000462000
	N	3.276345000	1.145761000	-0.793963000
	H	4.381042000	1.252614000	-0.898106000
	H	2.922374000	2.050188000	-0.272338000
	N	3.276290000	-1.260187000	-0.596426000
	H	4.380988000	-1.403636000	-0.637475000
	H	2.921770000	-1.260569000	-1.640296000
	N	3.277287000	0.113862000	1.388439000
	H	4.382073000	0.150501000	1.532358000
	H	2.923549000	-0.790098000	1.911032000
	N	-3.301745000	-0.885673000	1.073424000
	H	-4.404528000	-1.016309000	1.131174000
	H	-2.997096000	-0.421379000	2.027002000
	N	-3.302293000	-0.485675000	-1.302682000
	H	-4.405122000	-0.470083000	-1.444337000
	H	-2.997936000	-1.543692000	-1.377639000
	N	-3.301764000	1.371975000	0.231793000
	H	-4.404513000	1.487186000	0.316727000
	H	-2.997746000	1.965730000	-0.647240000
	N	2.665236000	1.155498000	-2.176268000
	N	1.337953000	1.090111000	-2.267129000
	N	3.478702000	1.250903000	-3.311907000
	N	0.757875000	1.098360000	-3.464662000
	N	2.881007000	1.264338000	-4.567687000
	N	1.496920000	1.180012000	-4.654214000
	H	4.564598000	1.301956000	-3.207272000
	H	3.489213000	1.323006000	-5.473272000
	H	1.009193000	1.150578000	-5.629210000
	N	-0.728878000	0.990400000	-3.493200000
	N	-1.336068000	0.422422000	-2.440849000
	N	-1.459517000	1.451208000	-4.596985000
	N	-2.663160000	0.250868000	-2.456662000

	N -2.838357000 1.296653000 -4.609371000<br>N -3.453039000 0.678309000 -3.526340000<br>H -0.953994000 1.946271000 -5.426166000<br>H -3.429589000 1.659539000 -5.453245000<br>H -4.534479000 0.530900000 -3.501159000<br>N 2.666717000 1.306176000 2.088336000<br>N 1.339474000 1.418028000 2.077216000<br>N 3.480590000 2.241716000 2.738613000<br>N 0.759792000 2.451184000 2.683158000<br>N 2.883323000 3.322777000 3.378135000<br>N 1.499272000 3.440330000 3.348509000<br>H 4.566447000 2.125226000 2.730323000<br>H 3.491831000 4.077529000 3.881630000<br>H 1.011927000 4.299625000 3.810531000<br>N -0.726956000 2.530347000 2.604241000<br>N -1.334748000 1.902936000 1.586531000<br>N -1.457012000 3.256374000 3.555260000<br>N -2.661843000 2.003076000 1.446240000<br>N -2.835831000 3.345074000 3.427967000<br>N -3.451111000 2.716401000 2.351260000<br>H -0.951065000 3.726826000 4.398388000<br>H -3.426577000 3.894914000 4.164213000<br>H -4.532559000 2.768857000 2.211276000<br>N 2.665600000 -2.462346000 0.086327000<br>N 1.338376000 -2.508343000 0.189109000<br>N 3.479329000 -3.493665000 0.570796000<br>N 0.758576000 -3.549764000 0.780642000<br>N 2.881943000 -4.588175000 1.186897000<br>N 1.497907000 -4.621003000 1.303854000<br>H 4.565177000 -3.428493000 0.473791000<br>H 3.490351000 -5.401938000 1.588230000<br>H 1.010455000 -5.450918000 1.816684000<br>N -0.728137000 -3.520553000 0.889193000<br>N -1.335481000 -2.325261000 0.855063000<br>N -1.458595000 -4.706879000 1.042699000<br>N -2.662463000 -2.253263000 1.012549000<br>N -2.837319000 -4.640426000 1.183869000<br>N -3.452111000 -3.393380000 1.178167000<br>H -0.953042000 -5.672474000 1.028259000<br>H -3.428383000 -5.552716000 1.292191000<br>H -4.533462000 -3.297941000 1.294137000<br>H -0.464982000 0.735726000 0.598387000<br>H -0.465252000 -0.886717000 0.338039000<br>H -0.465333000 0.150045000 -0.936769000<br>O -0.120238000 -0.000377000 -0.000179000
[K⊂[2.2.2]cryptand]	PBE0/def2-SVP (0 imaginary Frequencies)
	O 1.409145000 2.389453000 0.528834000

	O	-1.410152000	2.433294000	0.260529000
	O	-1.410260000	-0.991887000	-2.235629000
	O	1.409801000	-0.735147000	-2.333183000
	O	1.410704000	-1.654239000	1.801948000
	O	-1.409083000	-1.443275000	1.975532000
	N	-3.080228000	0.000651000	0.000852000
	N	3.079921000	0.001267000	0.000327000
	N	2.755626000	-1.361768000	2.075540000
	H	3.229108000	-2.211515000	2.607688000
	H	2.829145000	-0.488057000	2.753208000
	N	3.512959000	-1.144216000	0.784869000
	H	4.598350000	-1.085304000	1.026058000
	H	3.379447000	-2.053604000	0.181042000
	N	2.754881000	2.480010000	0.141543000
	H	3.227300000	3.365933000	0.612083000
	H	2.830868000	2.629615000	-0.953834000
	N	3.511364000	1.253764000	0.600484000
	H	4.597090000	1.433376000	0.431135000
	H	3.375442000	1.185711000	1.689696000
	N	2.755264000	-1.116270000	-2.216545000
	H	3.228424000	-1.153157000	-3.218643000
	H	2.830227000	-2.139219000	-1.797079000
	N	3.511547000	-0.105190000	-1.384440000
	H	4.597319000	-0.341422000	-1.455201000
	H	3.375436000	0.871892000	-1.870530000
	N	0.689160000	-2.027904000	2.946252000
	H	0.614783000	-1.179580000	3.655119000
	H	1.201057000	-2.859506000	3.472216000
	N	-0.687465000	-2.494420000	2.562156000
	H	-0.612984000	-3.353046000	1.865803000
	H	-1.199389000	-2.850875000	3.479257000
	N	0.687800000	3.566746000	0.277137000
	H	0.613232000	3.753579000	-0.812462000
	H	1.199943000	4.439173000	0.731885000
	N	-0.688672000	3.469184000	0.873821000
	H	-0.613851000	3.299168000	1.966143000
	H	-1.200867000	4.441008000	0.720738000
	N	0.688247000	-1.543844000	-3.224879000
	H	0.614316000	-2.580093000	-2.839626000
	H	1.199920000	-1.588191000	-4.207957000
	N	-0.688634000	-0.979470000	-3.439326000
	H	-0.614544000	0.051132000	-3.839374000
	H	-1.200336000	-1.599118000	-4.203806000
	N	-2.755263000	2.394658000	0.658580000
	H	-3.228423000	3.384283000	0.496735000
	H	-2.829272000	2.186202000	1.744438000
	N	-3.512620000	1.379043000	-0.166992000
	H	-4.598053000	1.494594000	0.052564000

	H -3.378569000 1.662653000 -1.221051000<br>N -2.754677000 -1.767117000 1.743900000<br>H -3.227349000 -2.122233000 2.681940000<br>H -2.830055000 -2.602431000 1.019654000<br>N -3.511236000 -0.543184000 1.278999000<br>H -4.596995000 -0.789729000 1.271001000<br>H -3.374810000 0.227252000 2.051868000<br>N -2.755085000 -0.626776000 -2.401167000<br>H -3.228630000 -1.261050000 -3.177613000<br>H -2.828131000 0.418074000 -2.763052000<br>N -3.512571000 -0.834063000 -1.108892000<br>H -4.598017000 -0.701885000 -1.318713000<br>H -3.378248000 -1.888742000 -0.827728000<br>K -0.000302000 0.000578000 -0.000124000
[K^{+}⊂[2.2.2]cryptand]]	PBE0/def2-SVP (0 imaginary Frequencies)
	O 1.413266000 2.456468000 0.131320000<br>O -1.414338000 2.458952000 -0.122907000<br>O -1.414308000 -1.335631000 -2.067860000<br>O 1.413394000 -1.114409000 -2.193070000<br>O 1.413416000 -1.342105000 2.061712000<br>O -1.414131000 -1.123360000 2.190240000<br>N -3.081137000 -0.000059000 0.000158000<br>N 3.080405000 -0.000191000 0.000032000<br>N 2.749288000 -0.967291000 2.286134000<br>H 3.234816000 -1.683610000 2.977218000<br>H 2.791229000 0.023710000 2.779637000<br>N 3.518460000 -0.980594000 0.983781000<br>H 4.599487000 -0.866277000 1.218055000<br>H 3.400151000 -1.986140000 0.554130000<br>N 2.749220000 2.463348000 -0.305232000<br>H 3.234747000 3.419949000 -0.030249000<br>H 2.791452000 2.395313000 -1.410188000<br>N 3.518205000 1.341980000 0.357456000<br>H 4.599310000 1.487816000 0.141740000<br>H 3.399465000 1.472397000 1.443127000<br>N 2.749453000 -1.495882000 -1.981278000<br>H 3.234798000 -1.735286000 -2.947490000<br>H 2.792177000 -2.419277000 -1.370644000<br>N 3.518239000 -0.361435000 -1.341107000<br>H 4.599400000 -0.620854000 -1.359633000<br>H 3.399071000 0.513687000 -1.996637000<br>N 0.682768000 -1.520951000 3.247760000<br>H 0.584443000 -0.562278000 3.794530000<br>H 1.198567000 -2.235720000 3.918020000<br>N -0.682738000 -2.065990000 2.931028000<br>H -0.584268000 -3.014898000 2.367441000<br>H -1.198004000 -2.296117000 3.883794000

	N 0.682858000 3.573397000 -0.306282000<br>H 0.584751000 3.568362000 -1.409921000<br>H 1.198750000 4.510968000 -0.021660000<br>N -0.682768000 3.571359000 0.323863000<br>H -0.584399000 3.556734000 1.427425000<br>H -1.197830000 4.511867000 0.047481000<br>N 0.682820000 -2.052069000 -2.941114000<br>H 0.584646000 -3.005104000 -2.384544000<br>H 1.198580000 -2.274796000 -3.895354000<br>N -0.682737000 -1.505300000 -3.254573000<br>H -0.584245000 -0.542531000 -3.794132000<br>H -1.197886000 -2.215079000 -3.930638000<br>N -2.749170000 2.461879000 0.316994000<br>H -3.236210000 3.419773000 0.049188000<br>H -2.788736000 2.386995000 1.421634000<br>N -3.518697000 1.343881000 -0.350596000<br>H -4.599680000 1.488990000 -0.133812000<br>H -3.400279000 1.479570000 -1.435640000<br>N -2.749068000 -1.505653000 1.973327000<br>H -3.235741000 -1.752683000 2.936968000<br>H -2.788942000 -2.424769000 1.356049000<br>N -3.518877000 -0.368507000 1.339274000<br>H -4.599754000 -0.629302000 1.356248000<br>H -3.401082000 0.503259000 1.999543000<br>N -2.749114000 -0.956178000 -2.290487000<br>H -3.236002000 -1.667003000 -2.986287000<br>H -2.788624000 0.037965000 -2.777861000<br>N -3.518876000 -0.975512000 -0.988594000<br>H -4.599767000 -0.859767000 -1.222883000<br>H -3.401179000 -1.983261000 -0.563871000<br>K 0.001471000 -0.000160000 0.000015000
[Na⊂[bpy.bpy.bpy]cryptand]	PBE0/def2-SVP (0 imaginary Frequencies)
	Na -0.000422000 -0.150929000 -0.001192000<br>N -1.655051000 0.028805000 2.235855000<br>N 1.654991000 0.045615000 -2.235765000<br>N 2.724978000 0.966025000 -1.958941000<br>H 3.381510000 1.136584000 -2.843800000<br>H 3.369055000 0.515192000 -1.184245000<br>N 0.800903000 0.484231000 -3.315497000<br>H 1.340144000 0.511638000 -4.289379000<br>H 0.485514000 1.518784000 -3.100398000<br>N 2.123780000 -1.311564000 -2.404794000<br>H 2.839838000 -1.405834000 -3.251978000<br>H 1.253038000 -1.938021000 -2.658710000<br>N -2.133803000 -1.326855000 2.387957000<br>H -2.851068000 -1.426335000 3.233535000<br>H -1.267841000 -1.962740000 2.634701000<br>N -2.718204000 0.960412000 1.970387000

	H	-3.374138000	1.124148000	2.856976000
	H	-3.364903000	0.524383000	1.189412000
	N	-0.797717000	0.447716000	3.320863000
	H	-1.336776000	0.467411000	4.295022000
	H	-0.474298000	1.482320000	3.118274000
	N	2.271797000	2.306327000	-1.433988000
	N	1.108536000	2.366183000	-0.794134000
	N	3.086705000	3.434074000	-1.603162000
	N	0.680368000	3.533995000	-0.308200000
	N	2.669042000	4.644066000	-1.066084000
	N	1.443742000	4.703577000	-0.409847000
	H	4.032400000	3.351694000	-2.143731000
	H	3.289525000	5.538655000	-1.160258000
	H	1.097700000	5.639641000	0.029966000
	N	-0.654077000	3.534622000	0.354127000
	N	-1.091114000	2.363708000	0.824448000
	N	-1.408666000	4.708452000	0.471201000
	N	-2.254936000	2.304123000	1.463321000
	N	-2.634475000	4.649481000	1.126528000
	N	-3.061326000	3.435637000	1.647466000
	H	-1.055517000	5.647620000	0.043834000
	H	-3.248210000	5.547392000	1.232547000
	H	-4.007683000	3.353213000	2.186867000
	N	2.759387000	-1.880263000	-1.158914000
	N	2.211364000	-1.541081000	0.004949000
	N	3.859443000	-2.733025000	-1.270744000
	N	2.745557000	-2.015984000	1.155998000
	N	4.393769000	-3.270873000	-0.091573000
	N	3.841636000	-2.909661000	1.121742000
	H	4.280738000	-2.970365000	-2.249594000
	H	5.241657000	-3.959528000	-0.127764000
	H	4.253612000	-3.321957000	2.042753000
	N	2.146355000	-1.571452000	2.414216000
	N	1.034269000	-0.801370000	2.342547000
	N	2.696427000	-1.921431000	3.670413000
	N	0.440456000	-0.405070000	3.465363000
	N	2.082814000	-1.492910000	4.830533000
	N	0.913329000	-0.725296000	4.739558000
	H	3.604478000	-2.520889000	3.733187000
	H	2.500325000	-1.758251000	5.805110000
	H	0.381825000	-0.386188000	5.630824000
	N	-0.443910000	-0.356981000	-3.470474000
	N	-1.039844000	-0.764261000	-2.352734000
	N	-0.920432000	-0.655693000	-4.748551000
	N	-2.157945000	-1.524567000	-2.434057000
	N	-2.096044000	-1.412624000	-4.849068000
	N	-2.711983000	-1.852508000	-3.694421000

	H -0.387077000 -0.308349000 -5.635532000<br>H -2.516628000 -1.660819000 -5.826837000<br>H -3.625034000 -2.443495000 -3.764594000<br>N -2.759602000 -1.981851000 -1.181554000<br>N -2.222450000 -1.525119000 -0.024554000<br>N -3.860976000 -2.869321000 -1.158766000<br>N -2.772722000 -1.875687000 1.134856000<br>N -4.415432000 -3.242618000 0.049843000<br>N -3.878074000 -2.722965000 1.235749000<br>H -4.275093000 -3.267555000 -2.084995000<br>H -5.267382000 -3.926650000 0.077241000<br>H -4.300975000 -2.970065000 2.211487000
[Na^+⊂[bpy.bpy.bpy]cryptand]]	PBE0/def2-SVP (0 imaginary Frequencies)
	Na 0.000232000 0.000758000 0.000453000<br>N 0.002931000 -0.002246000 2.791819000<br>N -0.002166000 0.002337000 -2.792129000<br>N 0.426358000 1.321625000 -3.193567000<br>H 0.363353000 1.470668000 -4.294159000<br>H 1.492268000 1.429122000 -2.931423000<br>N -1.359125000 -0.285355000 -3.193699000<br>H -1.457001000 -0.412501000 -4.294495000<br>H -1.984955000 0.583636000 -2.929853000<br>N 0.925280000 -1.028421000 -3.195625000<br>H 1.083462000 -1.048336000 -4.296563000<br>H 0.485830000 -2.005192000 -2.932512000<br>N 0.462689000 -1.311392000 3.192120000<br>H 0.405845000 -1.461853000 4.292851000<br>H 1.530105000 -1.394234000 2.927192000<br>N -1.359613000 0.253992000 3.195901000<br>H -1.458350000 0.378735000 4.296883000<br>H -1.965703000 -0.629205000 2.933110000<br>N 0.907117000 1.049329000 3.194375000<br>H 1.066718000 1.072104000 4.295053000<br>H 0.444929000 2.015951000 2.932731000<br>N -0.320952000 2.440409000 -2.508175000<br>N -0.821217000 2.203826000 -1.298008000<br>N -0.446095000 3.684078000 -3.138483000<br>N -1.488561000 3.171472000 -0.659913000<br>N -1.099655000 4.707578000 -2.464891000<br>N -1.635972000 4.451556000 -1.207461000<br>H -0.032775000 3.840138000 -4.137380000<br>H -1.198544000 5.697253000 -2.916789000<br>H -2.146528000 5.242509000 -0.657587000<br>N -2.068600000 2.825700000 0.667727000<br>N -1.538398000 1.775136000 1.303415000<br>N -3.120732000 3.567649000 1.217893000<br>N -1.984087000 1.447500000 2.513656000<br>N -3.600700000 3.217615000 2.475442000

	N	-3.014684000	2.152442000	3.146574000
	H	-3.571073000	4.395682000	0.669985000
	H	-4.421323000	3.777965000	2.929349000
	H	-3.348347000	1.862856000	4.145469000
	N	2.269276000	-0.941158000	-2.513138000
	N	2.317092000	-0.390789000	-1.302553000
	N	3.407485000	-1.454139000	-3.146349000
	N	3.490151000	-0.297337000	-0.666888000
	N	4.622030000	-1.400848000	-2.475193000
	N	4.671190000	-0.809536000	-1.217329000
	H	3.333770000	-1.889129000	-4.145513000
	H	5.527525000	-1.809839000	-2.929340000
	H	5.612606000	-0.763516000	-0.669376000
	N	3.483680000	0.377270000	0.661089000
	N	2.309994000	0.443798000	1.298969000
	N	4.653615000	0.916562000	1.209372000
	N	2.251601000	0.993193000	2.509525000
	N	4.593049000	1.506842000	2.467246000
	N	3.378810000	1.532407000	3.140578000
	H	5.594891000	0.892147000	0.659788000
	H	5.489708000	1.936640000	2.919709000
	H	3.296943000	1.965866000	4.139775000
	N	-1.954614000	-1.492857000	-2.510272000
	N	-1.499256000	-1.810130000	-1.300875000
	N	-2.969802000	-2.221354000	-3.141378000
	N	-2.003867000	-2.872718000	-0.664412000
	N	-3.529883000	-3.299804000	-2.469303000
	N	-3.039618000	-3.638752000	-1.212685000
	H	-3.311886000	-1.939527000	-4.139645000
	H	-4.338162000	-3.878937000	-2.921779000
	H	-3.469601000	-4.477021000	-0.664006000
	N	-1.413464000	-3.205040000	0.662064000
	N	-0.767153000	-2.222343000	1.298823000
	N	-1.530971000	-4.487967000	1.210221000
	N	-0.260063000	-2.447100000	2.508395000
	N	-0.987287000	-4.731288000	2.466998000
	N	-0.356230000	-3.692979000	3.139376000
	H	-2.024270000	-5.290503000	0.661371000
	H	-1.063104000	-5.722782000	2.919367000
	H	0.061778000	-3.839176000	4.137814000
